# Enhancing Electrical Generation Efficiency through Parametrical Excitation and Slapping Force in Nonlinear Elastic Beams for Vibration Energy Harvesting

**DOI:** 10.3390/s23177610

**Published:** 2023-09-01

**Authors:** Yi-Ren Wang, Chun-Hsiao Kuo

**Affiliations:** Department of Aerospace Engineering, Tamkang University, New Taipei City 25137, Taiwan; 611430074@o365.tku.edu.tw

**Keywords:** energy harvester, parametric excitation, nonlinear vibration, method of multiple scales

## Abstract

This study aims to enhance conventional vibration energy harvesting systems (VEHs) by repositioning the piezoelectric patch (PZT) in the middle of a fixed–fixed elastic steel sheet instead of the root, as is commonly the case. The system is subjected to an axial simple harmonic force at one end to induce transversal vibration and deformation. To further improve power conversion, a baffle is strategically installed at the point of maximum deflection, introducing a slapping force to augment electrical energy harvesting. Employing the theory of nonlinear beams, the equation of motion for this nonlinear elastic beam is derived, and the method of multiple scales (MOMS) is used to analyze the phenomenon of parametric excitation. This study demonstrates through experiments and theoretical analysis that the second mode yields better power generation benefits than the first mode. Additionally, the voltage generation benefits of the enhanced system with the added baffle (slapping force) surpass those of traditional VEH systems. Overall, the proposed model proves feasible and holds promising potential for efficient vibration energy harvesting applications in various industrial sectors.

## 1. Introduction

Advancements in technology and global population growth have catalyzed the growth of green energy, a pivotal aspect of the emerging industrial revolution. Within the realm of eco-friendly energy sources, vibration energy, despite its significance, often remains underexplored. Vibrations, prevalent in diverse settings from compact devices to substantial structures like buildings and vehicles, offer a potential yet untapped resource. A focal point of current research lies in vibrational energy harvesters (VEH) designed to efficiently harness this energy. The conventional approach involves utilizing a piezoelectric patch (PZT) on an elastic steel sheet to convert mechanical energy into electrical energy during sheet vibrations, known as single elastic-steel VEH systems (SES-VEHs). However, this configuration demands extended and pliable PZT patches, increasing costs while seeking enhanced efficiency. A novel alternative, double elastic-steel VEH systems (DES-VEHs), has been proposed by some researchers. This approach positions the PZT patch at the free end of a single elastic steel sheet, which is subsequently subjected to impact force from another sheet. The DES-VEH eliminates the need for elongated and delicate PZTs. Nonetheless, challenges persist in achieving optimal deformation and power generation simultaneously. The ongoing exploration of this domain exhibits the potential to revolutionize energy-harvesting methods, contributing to reduced environmental impact. This study aims to enhance the conventional vibration energy harvester (VEH) design by positioning a PZT on a fixed–fixed elastic steel sheet. Transverse vibration and deformation are induced by applying a simple harmonic force axially to one end of the fixed–fixed steel sheet. At the juncture of maximum deformation (possibly near the midpoint), a baffle is introduced to generate an impact force (slapping) on the PZT, thereby increasing energy conversion. Through the integration of the advantages of SES-VEH and DES-VEH, this approach achieves optimal power generation efficiency.

VEHs do not require external power and possess superior power–electricity coupling, making them compatible with microelectro-mechanical system (MEMS) fabrication. Moreover, their broad applicability is attributable to the lack of additional devices such as coils and magnets. The traditional method for applying piezoelectric energy involves accumulating energy before supplying power to other electronic devices. However, Sodano and Inman [1] broke through the traditional concept of piezoelectric materials by using a rechargeable battery to store the electric energy generated by piezoelectric materials. Compared to traditional methods, this approach can more directly provide power to electronic systems, laying the foundation for future wireless and microelectromechanical systems (MEMSs). Rajora et al. [2] proposed an analytical method for estimating the output of the amplitude, voltage, and power generated by the vibration of a fixed–free Euler–Bernoulli beam. The validity of their approach was tested using engineering simulation software ANSYS12 and multiphysics simulation software COMSOL 4.3. Masana and Daqaq [3] analyzed the vibration of a fixed–fixed beam subjected to axial forces. They used a nonlinear Bernoulli–Euler beam as a theoretical model, which was expanded using the Galerkin method. They employed the Method of Multiple Scales (MOMS) to obtain analytical expressions for the steady-state response amplitude, voltage under resistive load, and output power. They also analyzed the maximum transverse amplitude of the beam using a fixed-point plot and found that axial perturbations were able to provide the maximum transverse amplitude of this nonlinear beam. Li [4] investigated the vibration stability of a nonlinear sandwich beam with axial parameter perturbations, while Yan [5] studied a nonlinear Timoshenko beam with similar perturbations. Both studies utilized axial perturbations to excite the system, which is a typical parametric excitation problem. Lagrange’s equations and Galilean transformations were used in both studies to derive the equations of motion. Galerkin’s method was used to solve the equations of motion and obtain the system’s frequency response. The results showed that under specific parameter and external driving source perturbation conditions, the system may exhibit internal resonance or chaotic phenomena, leading to the multiplication of the amplitude and structural instability.

Shibata et al. [6] installed a linear spring attachment at the axial end of a nonlinear beam and used a longitudinal linear spring to control its transverse vibration. The spring only affected the nonlinear characteristics of the transverse vibration and did not impact the beam’s linear natural frequency. As a result, the unstable region in the parametric excitation remained unchanged. They conducted experiments to analyze the effectiveness of vibration reduction. Plat and Bucher [7] investigated the nonlinear string system of a parametric excitation vibration. The passive dynamical system significantly amplified the limited transverse vibration amplitude of the nonlinear string, thereby increasing the system’s selectivity. Bagheri et al. [8] used the Bernoulli–Euler theory model structure to examine the nonlinear response of a clamped–clamped bending beam. They applied axial loading at one end and used two numerical methods, He’s Variational Approach and the Laplace Iteration Method, to predict the lateral transverse beam vibration. They found that these two numerical methods could be applied to other nonlinear vibrations, and the vibration frequency of the nonlinear lateral transverse beam was affected by the axial load.

In recent years, there has been a surge in research on energy harvesting systems that rely mainly on impact force. Notably, several new studies were published almost simultaneously, demonstrating the growing importance of this field. One example is the work of Fu et al. [9], who used the electrostatic effect of collisions between three parallel cantilever beams to generate vibrations and electrical energy. Their research provides theoretical evidence that the impact effect can increase the power generation efficiency of specific materials. Around the same time, Wang and their team (Wang et al. [10,11]) also investigated the use of impact-induced vibrations to generate electricity. They not only established a theoretical model, but also demonstrated the feasibility of the concept whereby a piezoelectric patch could generate electricity by being impacted. Wang et al. [10] designed a double-layer elastic-steel-sheet energy harvesting system comprising two parallel cantilever beams (Double Elastic Steel VEH (DES-VEH)). Wang et al. [11], on the other hand, examined the energy conversion efficiency of transverse vibrations and mutual impacts of a group (two pieces) of Fixed–fixed beams. To optimize the impact force, the placement position of the piezoelectric patch (PZT) is crucial. The aforementioned studies [10,11] analyzed DES to identify its system peaks and nodes, determine the maximum deformation and optimal position of the PZT, and ultimately achieve the maximum power generation efficiency. Wang and Chu [12] utilized the airflow generated beneath the rotor of a rotary-wing aircraft to drive a wind turbine, creating a rotating magnetic field. This field then generated a repulsive force with a magnet placed on an elastic steel sheet, causing two elastic steel sheets to strike each other and the PZT, thereby producing vibrational energy and converting it into electrical energy. Meanwhile, Wang and Cheng [13] placed the elastic steel sheets in the middle of a rotor and utilized the rotating magnetic field to cause the two sheets (with PZTs) to clap each other. Through theoretical analysis and experiments, they demonstrated that the energy conversion efficiency of the system utilizing two elastic steel sheets exceeded that of traditional single-sheet systems. In addition, some scholars have proposed bistable vibration energy harvesting systems (BVEHs), which mainly consist of two types of system: cantilever beams and buckling beams. The cantilever beam is designed with one end fixed and the other end attached to a magnet. One or two magnets with the same polarity are placed next to the end to cause the cantilever beam to vibrate up and down under the influence of magnetic force, simulating a bistable system. The buckling beam, on the other hand, can achieve a bistable system without the use of magnets. The biggest advantage of BVEHs is that they can be driven in low-frequency and low-amplitude environments. Harne and Wang [14] and others have conducted research on the theoretical simulation and integrated analysis of the bistable systems of both beams.

Mokhtari et al. [15,16] provided a review of the current state of wearable energy harvesting technologies. The review highlighted challenges and potential solutions, introducing the concept of flexible-fiber energy harvesters as a promising avenue. The article’s focus on dimension conversion and the advantages of piezoelectric energy harvesting devices contributes to the understanding of the technology landscape. Costa et al. [17] explored the diverse field of flexible sensors and sensing systems. It delved into the mechanics, materials, and devices involved in creating flexible sensors. They provided a comprehensive understanding of how flexible sensors could be integrated into various systems, including wearable technologies, and their potential impact on enhancing sensing capabilities. Mokhtari et al. [18] introduced a novel wearable energy generator using hybrid piezoelectric nano fibers, achieving high power density and faster charging capabilities for real-time healthcare monitoring. The mentioned literature focused on flexible and nano sensors, while from a PZT power generation perspective, the PZT materials were chosen primarily for their ability to convert mechanical vibrations directly into electrical energy through the piezoelectric effect. They were optimized for energy harvesting applications and were strategically placed at points of maximum deformation to efficiently harness vibrations to achieve power generation. In addition, compared with flexible sensors or nano sensors, PZTs have the advantage of lower price, potentially making them suitable for industrial applications.

Zhu and Zu [19] attached piezoelectric materials to both ends of a fixed beam and placed a magnet at one end. They induced a small axial disturbance using electromagnetic induction to simulate low-frequency (<60 Hz) and small-oscillation (<0.5 g) scenarios. The experimental results showed that the voltage peaks generated by the conversion between the two energy wells were maintained at around 10 volts, demonstrating the potential of this system for future development. Xu et al. [20] used a bistable system for energy harvesting in microelectromechanical systems (MEMSs). Traditional vibration energy harvesting relies on achieving the resonance frequency in order to obtain a larger amplitude and power generation.

Derakhshani et al. [21] developed theoretical and experimental models for a fixed–fixed bistable buckling beam with driving frequencies below 30 Hz. Using Hamilton’s principle, they derived a coupling between the nonlinear Bernoulli–Euler beam and the piezoelectric equation to analyze the theoretical and experimental output voltages for different vibration scenarios. In addition, Derakhshani et al. [22] coupled a fixed–fixed beam with two fixed–fixed beams to establish a bistable bukling system. They disturbed the system using a torsional rod and two co-directional cantilever beams to facilitate fast switching between two stable regions and achieve better power generation. They also compared the power generation efficiency of PZTs placed in different locations, confirming the feasibility of this system for future engineering applications. Cottone et al. [23] employed bistable oscillators placed at one end of a fixed–fixed beam to simulate random vibrations. Using the Euler–Maruyama method, they compared the performance of the bistable energy wells at different levels of buckling and resistance and found that the system’s output power load increased with the amplitude of the system vibration when subjected to wide-bandwidth Gaussian noise. Marinca et al. [24] investigated the low-frequency buckling vibration and impact force of the bistable system using a double-layer-structure system composed of a primary buckled piezoelectric beam and a rubber sheet. They applied Hertzian Contact Force to one end and found that the theoretical power generation efficiency of the two forces combined was the same as that reported by Wang et al.’s research team [10,11,12,13], who demonstrated that the energy conversion efficiency of two elastic steel sheets slapping a PZT was better than that of traditional single elastic steel sheet energy harvesting systems. Osinaga et al. [25] analyzed the power generation efficiency of buckling beams before (monostable) and after (bistable) buckling. They coupled the nonlinear Bernoulli–Euler beam and piezoelectric equations to calculate the states of buckling beams before and after buckling, discretized the space using the Galerkin method, and found analytical expressions for the displacement amplitudes of each mode. Then, they used MOMS to find linear approximations of the pre- and post-buckling states. Du et al. [26] introduced a piezoelectric buckling-beam-type bistable energy harvester (PBBEH) for efficient energy extraction from rotational motions. The PBBEH integrates a piezoelectric buckling beam and a rotational disk to capture low-speed rotational movements. A lumped parameter model is used for numerical analysis and energy harvesting characteristics are examined. Experimental results highlight excellent performance within the 1–9 Hz frequency range, yielding output power of 28 μW. These conventional buckled VEH systems position the PZT at the elastic steel root, neglecting the middle placement crucial for effective slapping. Moreover, axial excitation’s parametric phenomenon, which is key to achieving broader bandwidth, is overlooked, prompting this study’s analysis of parametric excitation effects.

Mei et al. [27] introduced a clamped–clamped flexible piezoelectric energy harvester (FPEH) for enhanced power output and adaptability to low-frequency vibrations in wearable electronics. The harvester incorporated axial excitation and pre-deformation, and its dynamic equation was derived. Numerical analysis and experiments confirmed simulation alignment, showcasing promising LED power results: max output power 1.38 μW at 27 Hz; output voltage 1.84 V. However, their modes were constrained to a fixed resonant frequency, limiting wide-bandwidth application. Qin Y et al. [28] developed a distributed-parameter dynamic model for a fixed–fixed piezoelectric energy harvester using the Euler–Bernoulli beam hypothesis and Hamilton’s principle. Their model adjusted the system’s natural frequency via proof mass movement to broaden the frequency band and align with external excitation. Shim et al. [29] designed a nonlinear piezoelectric energy harvester with a coupled beam array, thus amplifying the bandwidth and energy harvesting via elastic supports that enhance nonlinear behavior. Experimental validation demonstrated 144.2% higher average output power and 93.3% wider bandwidth compared to non-coupled multi-resonance harvesters. However, employing multiple elastic beams is necessary to achieve a broader bandwidth in their model.

In addition, when buckling beams are applied to objects undergoing severe vibrations, the system is typically placed on the ground, and energy is generated by utilizing the weight of passing vehicles or crowds. Ansari et al. [30] proposed placing the buckling beam underground to directly bear the weight of vehicles or crowds and generate power through buckling. However, considering the possibility of beam fracture due to long-term use and excessive buckling amplitude, strain analysis of the primary buckling beam is necessary to determine the optimal buckling amplitude of the elastic beam, thus obtaining the best power generation efficiency. In summary, the conversion of vibration energy from buckling beams is one of the most effective designs in terms of power generation efficiency. However, Wang et al.’s [10,11,12,13] research suggests that applying impact force to the VEH system can have an additional effect. Therefore, this study combines various methods proposed in the domestic and foreign literature to design a vibration energy extraction system with buckling impact, which can further advance VEH research. Wang et al.’s most recent research [13] found that precise frequency disturbances in VEH systems can increase power generation efficiency by more than 2%, especially in nonlinear systems. Therefore, conducting a comprehensive frequency parametric excitation analysis of the buckling beam system would be beneficial for assessing the system’s development efficiency.

**Figure 1:**
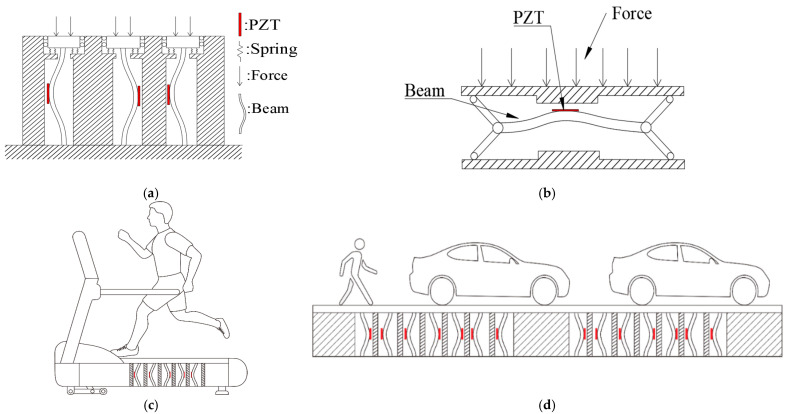
Application of the buckling beam VEH system: (**a**) vertical mode; (**b**) horizontal mode; (**c**) treadmill; (**d**) road.

Drawing from the discussed theoretical models, numerical analyses, and experimental applications, this study investigates the axial actuation amplitude and frequency of a nonlinear fixed–fixed Euler–Bernoulli beam. It analyzes the beam’s vibration stability and power generation efficiency across different modes and amplitudes, introducing an additional baffle to induce slapping force. A comparative assessment of power generation efficiency with and without the supplementary baffle aims to identify the optimal input frequency and baffle placement for efficient slapping energy. This design offers dual operational modes: vertical placement into the ground (depicted in Figure 1a) or a parallel arrangement (illustrated in Figure 1b). Both configurations find a variety of applications, spanning fitness equipment like treadmills (Figure 1c) and infrastructure such as sidewalks, roadways (Figure 1d), and railways.

This study consists of two main parts: theoretical simulation and experimental verification, aiming to explore the efficiency of converting vibration energy into electrical energy using a PZT attached to a fixed–fixed elastic steel sheet. In the theoretical portion, a nonlinear equation is derived using Newton’s Second Law, Euler’s angle transformation, and Taylor series expansion. The application of axial disturbances at the endpoints exemplifies a typical form of parametric excitation, and the Method of Multiple Scales (MOMS) is employed to analyze this phenomenon.

Analyzing parametric excitation led to the determination of the system’s unstable range, revealing the benefits of energy harvesting. The unstable range was validated through numerical analysis and fixed-point plots. Furthermore, the accuracy of the fixed-point plots was confirmed through the application of the fourth-order Runge–Kutta (RK-4) numerical method to generate time–response and phase plots. The maximization of the benefits of electrical energy conversion was achieved by combining the nonlinear equation with the piezoelectric equation while varying external forces and frequencies.

In the second segment of this study, a simple experiment was conducted using an elastic steel sheet to replicate the behavior of an elastic beam. Fixed boundary conditions for the beam’s ends were established using a C-shaped device. One end of the beam was held stationary, while the other end featured a horizontal sliding track and an actuator to induce vibration disturbances imitating buckling behavior. The experiment comprised two groups. Initially, the point of maximum deformation was identified using the elastic beam’s mode shape. Subsequently, a comparison was made between the power generation efficiency of piezoelectric patches placed at the root and at the location of maximum deformation. Subsequent to this, a piezoelectric patch was positioned at the point of maximum deformation, and a baffle was introduced to enhance the impact force. This facilitated the determination of the highest power generation efficiency and the validation of the accuracy of the theoretical model.

The rapid growth of wearable technologies calls for innovative advancements in energy generation systems. This work responds to this demand by introducing a unique approach to vibration energy harvesting. This study addresses the limitations of existing methods by strategically positioning a piezoelectric patch at the peak deformation point of an elastic steel sheet. This deliberate placement optimizes energy conversion efficiency by harnessing the maximum mechanical stress during vibrations. Additionally, the integration of an augmenting baffle introduces a novel element that amplifies the voltage generation capabilities of the system. By exploring the distinct advantages of the second mode and investigating the impact of the baffle, this research unveils new dimensions in vibration energy harvesting effectiveness. This study additionally investigates the frequency response of parametric excitation, extending the resonant frequency range around the linear natural frequency. This expansion contributes to a broader usable bandwidth compared to conventional designs.

## 2. Establishment and Analysis of the Theoretical Models

**Figure 2:**
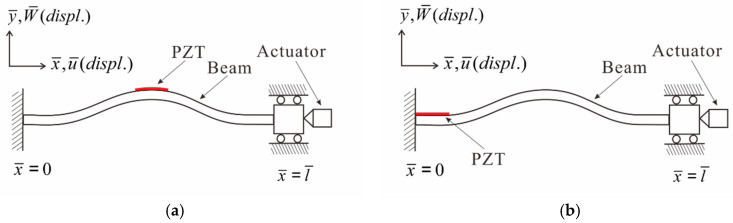
Fixed–fixed beam two-dimensional theoretical model: (**a**) PZT at maximum amplitude; (**b**) PZT at the root.

This study employs a nonlinear Euler–Bernoulli beam as the theoretical model. The elastic beam is fixed at one end, while the other end contains a roller allowing horizontal sliding. To simulate the buckling phenomenon, an actuator is positioned at the slidable end of the beam. This study investigates the buckling behavior of the elastic beam by applying external forces and varying the frequency. To evaluate power generation efficiency, piezoelectric patches (PZTs) are strategically placed at two specific locations along the beam: the point of maximum deformation and the root. A comparative analysis is conducted to determine the effectiveness of these locations in generating power. For a more comprehensive understanding of the coordinate definition and the composition of the two-dimensional theoretical model, please refer to Figure 2a,b. These figures provide detailed visual representations of the setup and configurations used in the study.

### 2.1. The Equation of Motion

Based on the nonlinear beam theory of Neyfeh and Pai [31], the two-dimensional nonlinear beam equation was formulated through the application of Newton’s Second Law, a three-dimensional Euler angle coordinate transformation, and Taylor expansion, as follows:

(1) $$\rho \ddot{\bar{u}}-EA{\bar{u}}^{\prime \prime }=EA(\frac{1}{2}{{\bar{W}}^{\prime }}^{2}-{\bar{u}}^{\prime }{{\bar{W}}^{\prime }}^{2}{)}^{\prime }+EI_{A}({\bar{W}}^{\prime }({\bar{W}}^{\prime \prime \prime }-{\bar{u}}^{\prime \prime \prime }{\bar{W}}^{\prime }-2{\bar{u}}^{\prime \prime }{\bar{W}}^{\prime \prime }-3{\bar{u}}^{\prime }{\bar{W}}^{\prime \prime \prime }){)}^{\prime }$$

(2) $$\begin{array}{l} \rho \ddot{\bar{W}}-EI_{A}{\bar{W}}^{iv}=EA({\bar{u}}^{\prime }{\bar{W}}^{\prime }-{{\bar{u}}^{\prime }}^{2}{\bar{W}}^{\prime }+\frac{1}{2}{{\bar{W}}^{\prime }}^{3})+EI_{A}[{\bar{u}}^{\prime }{\bar{W}}^{\prime \prime \prime }+({\bar{u}}^{\prime }{\bar{W}}^{\prime }{)}^{\prime \prime }-({{\bar{u}}^{\prime }}^{2}-{{\bar{W}}^{\prime }}^{2}){\bar{W}}^{\prime \prime \prime }-{\bar{u}}^{\prime }({\bar{u}}^{\prime }{\bar{W}}^{\prime }{)}^{\prime \prime }\\ -({\bar{u}}^{\prime }{\bar{W}}^{\prime }-\frac{1}{3}{{\bar{W}}^{\prime }}^{3}{)}^{\prime \prime }{]}^{\prime } \end{array}$$

where ρ is the density of the beam, *E* is the Young’s modulus of the beam, *A* is the cross-sectional area of the beam, *I* is the moment of inertia of the beam, $\overset{•}{\left(\;\right)}$ represents the differential with respect to time, and ( )**’** represents the differential with respect to $\bar{x}$ (i.e., $d/d\bar{x}$). Since a slender elastic beam is considered as the theoretical model, *m/EA* is extremely small. After dividing Equation (1) by *EA*, the longitudinal inertial force $m\ddot{\bar{u}}$ in Equation (1) can be ignored. In this study, the boundary conditions of the beam are considered to be fixed at both ends, and there is no lateral external force, so there will be no movement in the $\bar{u}$ direction. Then, the relationship between $\bar{u}$ and $\bar{W}$ is determined according to the boundary conditions, following which the equations can be simplified to a single $\bar{W}$-D.O.F. equation.

The following boundary conditions are assumed:

(3) $$\bar{u}(0,\bar{t})=0,\;\bar{u}(\bar{l},\bar{t})=\bar{P}(\bar{t}),\;\bar{W}(0,\bar{t})=0,\;\bar{W}(\bar{l},\bar{t})=0,\;{\bar{W}}^{\prime }(0,\bar{t})=0,\;{\bar{W}}^{\prime }(\bar{l},\bar{t})=0$$

where $\bar{P}(\bar{t})$ is the axial disturbance from the actuator. Substituting the boundary conditions (Equation (3)) into Equation (1), the $\bar{u}$-direction equation can be obtained as follows:

(4) $${\bar{u}}^{\prime \prime }=-(\frac{1}{2}{{\bar{W}}^{\prime }}^{2}{)}^{\prime }+({\bar{u}}^{\prime }{{\bar{W}}^{\prime }}^{2}{)}^{\prime }+\frac{\rho }{EA}\ddot{\bar{u}}+\frac{I_{A}}{A}[{\bar{W}}^{\prime }({\bar{W}}^{\prime \prime \prime }-{\bar{u}}^{\prime \prime \prime }{\bar{W}}^{\prime }-2{\bar{u}}^{\prime \prime }{\bar{W}}^{\prime \prime }-3{\bar{u}}^{\prime }{\bar{W}}^{\prime \prime \prime }){]}^{\prime }$$

after integrating twice, $\bar{u}$ can be found as:

(5) $$\bar{u}=\frac{1}{2}\int_{0}^{\bar{l}}{{\bar{W}}^{\prime }}^{2}d\bar{x}+c_{1}(\bar{t})\bar{x}+c_{2}(\bar{t})$$

The coefficients *c*_1_ and *c*_2_ can be obtained from the boundary conditions (Equation (3)) as $c_{2}(\bar{t})=0,\;c_{1}(\bar{t})=\frac{1}{2\bar{l}}\int_{0}^{\bar{l}}{{\bar{W}}^{\prime }}^{2}d\bar{x}$. Putting them into Equation (2), and adding the structural damping item $\bar{\mu }\dot{\bar{W}}$ of the elastic beam, using Newton’s Second Law, the nonlinear beam equation in $\bar{W}$ D.O.F. can be obtained as follows:

(6) $$\rho A\ddot{\bar{W}}+EI{\bar{W}}^{iv}+\bar{\mu }\dot{\bar{W}}=\frac{EA}{2\bar{l}}{\bar{W}}^{\prime \prime }\int_{0}^{1}{{\bar{W}}^{\prime }}^{2}d\bar{s}+EA\bar{P}(\bar{t}){\bar{W}}^{\prime \prime }$$

In order to make the research convenient for future analysis, the dimensionless form of Equation (4) can be obtained by firstly dividing equation (4) by ρA:

(7) $$\ddot{\bar{W}}+\frac{EI}{\rho A}{\bar{W}}^{iv}+\frac{\bar{\mu }}{\rho A}\dot{\bar{W}}=\frac{E}{2\bar{l}\rho }{\bar{W}}^{\prime \prime }\int_{0}^{1}{{\bar{W}}^{\prime }}^{2}d\bar{s}+\frac{E}{\rho }\bar{P}(\bar{t}){\bar{W}}^{\prime \prime }$$

and intorducing the following definitions: $W=\bar{W}/\bar{l}$, $t=\bar{t}\bar{\omega }$, $s=\sqrt{x^{2}+y^{2}}=\bar{s}/\bar{l}$, $x=\bar{x}/\bar{l}$, $\bar{\omega }={\left(\frac{EI}{\rho A{\bar{l}}^{4}}\right)}^{\frac{1}{2}}$, $l=\bar{l}/\bar{l}=1$, $\tilde{A}=A{\bar{l}}^{2}/I$, $\mu =\frac{\bar{\mu }{\bar{l}}^{2}}{EI}{\left(\frac{EI}{\rho A}\right)}^{\frac{1}{2}}$. Substituting these into Equation (5), the dimensionless nonlinear equation can be obtained as follows:

(8) $$\ddot{W}+W^{iv}+\mu \dot{W}=\frac{\tilde{A}}{2}W^{\prime \prime }\int_{0}^{1}{W^{\prime }}^{2}ds+\;\tilde{A}P(t)W^{\prime \prime }$$

### 2.2. Theoretical Analysis of the Piezoelectric Patch (PZT) Equation

From the research of Rajora et al. [2], it is known that the current equation for PZTs can be expressed as follows:

(9) $$C_{p}\dot{V}+\frac{1}{{\bar{R}}_{p}}V+\int_{\bar{a}}^{\bar{b}}eh_{p}t_{h}{\dot{\bar{W}}}^{\prime \prime }d\bar{x}=0$$

The force acting on the beam by the PZT is expressed as:

(10) $$\int_{\bar{a}}^{\bar{b}}eh_{p}t_{h}{\dot{\bar{W}}}^{\prime \prime }d\bar{x}=(et_{h}\int_{\bar{a}}^{\bar{b}}{\bar{W}}^{\prime \prime }d\bar{x})V=C_{f}(\int_{\bar{a}}^{\bar{b}}{\bar{W}}^{\prime \prime }d\bar{x})V$$

where $\bar{V}$ is the voltage, *C_f_* is the piezoelectric coupling coefficient, and *C_p_* is the capacitance of the piezoelectric patch. $\bar{a}$ and $\bar{b}$ are the positions of the two ends of the PZT, respectively. For example, if the PZT is placed at the root of the beam, and the length of the PZT is *P_l_* (that is, $\bar{b}$ − $\bar{a}$ = *P_l_*), then $\bar{a}$ = 0, $\bar{b}$ = *P_l_*. Dividing Equation (9) by $(m_{b}+m_{f})\bar{l}\omega_{u}^{2}$ provides the dimensionless PZT equation, as follows:

(11) $$\dot{\nu }+R_{p}\nu +\hat{k}\int_{a}^{b}{\dot{W}}^{\prime \prime }dx=0$$

where $v=V/C_{f}$, $R_{p}=1/{\bar{R}}_{p}C_{p}\bar{\omega }$, $\hat{k}=eh_{p}t_{h}/C_{p}C_{f}$, ${\left(\;\right)}^{*}=d/dt$, $(\;{)}^{\prime }=d/dx$, $a=\bar{a}/\bar{l}$, $b=\bar{b}/\bar{l}$. The dimensionless voltage can be obtained as follows:

(12) $$\nu =-\frac{\hat{k}}{e^{R_{p}t}}\int_{0}^{t}(\int_{a}^{b}{\dot{W}}^{\prime \prime }dx)e^{R_{p}t}dt$$

Dividing Equation (10) by $\bar{m}\bar{l}\;{\bar{\omega }}_{}^{2}$ and rearranging the terms yields the dimensionless external force (Coulomb force) function as follows:

(13) $$\frac{C_{f}^{2}(\int_{a}^{b}W^{\prime \prime }dx)\nu }{\bar{l}\bar{m}{\bar{\omega }}^{2}}=\eta^{2}(\int_{a}^{b}W^{\prime \prime }dx)(-\frac{\hat{k}}{e^{R_{p}t}}\int_{0}^{t}(\int_{a}^{b}{\dot{W}}^{\prime \prime }dx)e^{R_{p}t}dt)$$

where $\eta^{2}=C_{f}^{2}/\bar{l}\bar{m}{\bar{\omega }}^{2}$. Finally, the dimensionless nonlinear beam equation with the PZT patch can be obtained as follows:

(14) $$\ddot{W}+W^{iv}+\mu \dot{W}-\tilde{A}P(t)W^{\prime \prime }-\eta^{2}(\int_{a}^{b}W^{\prime \prime }dx)v=\frac{1}{2}\tilde{A}[\int_{0}^{1}{W^{\prime }}^{2}dx]W^{\prime \prime }$$

### 2.3. Method of Multiple Scales (MOMS)

The MOMS divides time into two scales—fast and slow; let $T_{0}=t$, $T_{1}=\varepsilon^{1}t$ and $T_{2}=\varepsilon^{2}t$, where $T_{0}$ is the term of the fast time scale, the other two items are for the slow time scale, and ε is the perturbation term, which is regarded as a very small value. *W* can be expressed as:

(15) $$W(x,t,\varepsilon )=\varepsilon W_{0}(x,T_{0},T_{1},T_{2},\ldots )+\varepsilon^{2}W_{1}(x,T_{0},T_{1},T_{2},\ldots )+\varepsilon^{3}W_{2}(x,T_{0},T_{1},T_{2},\ldots )$$

The end-point axial force is assumed to be a time-varying function, which can be expressed as $P_{0}+P(t)$, where $P(t)=Z\varepsilon^{2}\cos\Omega_{m}T_{0}$. The damping term of the elastic beam is $\varepsilon^{2}\mu$, and the influence of higher-order terms such as $\varepsilon^{4}$, $\varepsilon^{5}$, …, etc., on the system is ignored to facilitate subsequent analysis. Then, Equation (14) can be expressed as three time-scale (ε, $\varepsilon^{2}$ and $\varepsilon^{3}$) equations, as follows:

The ε-order time scale terms:

(16) $$\frac{\partial^{2}W_{0}}{\partial T_{0}^{2}}+W_{0}{}^{iv}-\tilde{A}P_{0}W_{0}^{\prime \prime }=0$$

The $\varepsilon^{2}$-order time scale terms:

(17) $$\frac{\partial^{2}W_{1}}{\partial T_{0}^{2}}+W_{1}{}^{iv}-\tilde{A}P_{0}W_{1}^{\prime \prime }=-2\frac{\partial^{2}W_{0}}{\partial T_{0}\partial T_{1}}+\tilde{A}P(t)W_{0}^{\prime \prime }$$

The $\varepsilon^{3}$-order time scale terms:

(18) $$\frac{\partial^{2}W_{2}}{\partial T_{0}^{2}}+W_{2}{}^{iv}-\tilde{A}P_{0}W_{2}^{\prime \prime }=-2\frac{\partial^{2}W_{1}}{\partial T_{0}\partial T_{1}}-\frac{\partial^{2}W_{0}}{\partial T_{1}^{2}}-2\frac{\partial^{2}W_{0}}{\partial T_{0}\partial T_{2}}-\mu \frac{\partial W_{0}}{\partial T_{0}}+\tilde{A}P(t)W_{1}^{\prime \prime }$$

The separation of variables method is used to find the mode shape of the system. The lateral deformation *W*_0_ is divided into the spatial and time domains, and defined as:

(19) $$W_{0}(x)=X(x)Y(t)$$

Substituting Equation (19) into Equation (16) gives:

(20) $$X\ddot{Y}+X^{iv}Y-\tilde{A}P_{0}X^{\prime \prime }Y=0$$

Using the boundary conditions, the characteristic equation of the elastic beam can be obtained as follows:

(21) $$2-2\cos(\sqrt{\alpha_{m}})-\alpha \sin(\sqrt{\alpha_{m}})=0$$

where $\alpha_{m}=4m^{2}\pi^{2}$ and *m* = 1, 2, 3, …. The mode shapes of the elastic beam can then be obtained as follows:

(22) $$X_{m}(x)=\sqrt{\frac{4(P_{0}-4m^{2}\pi^{2})}{2(m^{2}\pi^{2})}}[1-\cos(2m\pi x)]$$

## 3. System Parametric Excitation Analysis

### 3.1. Analysis of Stable and Unstable Regions

By defining $W_{0}(x)=\sum_{m=1}^{N}\phi_{m}(x)\xi_{0m}(t)$, $W_{1}(x)=\sum_{m=1}^{N}\phi_{m}(x)\xi_{1m}(t)$, and $W_{2}(x)=\sum_{m=1}^{N}\phi_{m}(x)\xi_{2m}(t)$, and substituting them into Equations (16)–(18) and applying the orthogonal method, the following equations are obtained:

The ε-order time scale terms:

(23) $${\ddot{\xi }}_{0m}(t)+\frac{\int_{0}^{1}\phi_{m}{}^{iv}\phi_{m}-\tilde{A}P_{0}\int_{0}^{1}\phi_{m}{}^{\prime \prime }\phi_{m}}{\int_{0}^{1}\phi_{m}{}^{2}}\xi_{0m}(t)=0$$

The $\varepsilon^{2}$-order time scale terms:

(24) $${\ddot{\xi }}_{1m}(t)+\frac{\int_{0}^{1}\phi_{m}{}^{iv}\phi_{m}-\tilde{A}P_{0}\int_{0}^{1}\phi_{m}{}^{\prime \prime }\phi_{m}}{\int_{0}^{1}\phi_{m}{}^{2}}\xi_{1m}(t)=-2\frac{\partial^{2}}{\partial T_{0}\partial T_{1}}\xi_{0m}(t)+\tilde{A}P_{\left(t\right)}\frac{\int_{0}^{1}\phi_{m}^{\prime \prime }\phi_{m}}{\int_{0}^{l}\phi_{m}^{2}dx}\xi_{0m}$$

The $\varepsilon^{3}$-order time scale terms:

(25) $$\begin{array}{l} {\ddot{\xi }}_{2m}(t)+\frac{\int_{0}^{1}\phi_{m}{}^{iv}\phi_{m}-\tilde{A}P_{0}\int_{0}^{1}\phi_{m}{}^{\prime \prime }\phi_{m}}{\int_{0}^{1}\phi_{m}{}^{2}}\xi_{2m}(t)=-2\frac{\partial^{2}}{\partial T_{0}\partial T_{1}}\xi_{1m}(t)-\frac{\partial^{2}}{\partial T_{1}{}^{2}}\xi_{0m}-2\frac{\partial^{2}}{\partial T_{0}\partial T_{2}}\xi_{0m}(t)-\mu \frac{\partial }{\partial T_{0}}\xi_{0m}+\tilde{A}P_{\left(t\right)}\frac{\int_{0}^{1}\phi_{m}^{\prime \prime }\phi_{m}}{\int_{0}^{l}\phi_{m}^{2}dx}\xi_{1m}\\ +\frac{1\tilde{A}\int_{0}^{1}\phi_{m}^{\prime \prime }\phi_{m}}{2\int_{0}^{1}\phi_{m}{}^{2}}\xi_{0m}{}^{3}\int_{0}^{1}{\phi^{\prime }}^{2}{}_{m}dx \end{array}$$

where $P(t)=Z\cos(2T_{0})=Z\frac{e^{i2T_{0}}+e^{-i2T_{0}}}{2}$, and *Z* is the dimensionless amplitude of the axial disturbance.

System frequency is defined as $\omega_{m}{}^{2}=\frac{\int_{0}^{1}\phi_{m}{}^{iv}\phi_{m}-\tilde{A}P_{0}\int_{0}^{1}\phi_{m}{}^{\prime \prime }\phi_{m}}{\int_{0}^{1}\phi_{m}{}^{2}}=\delta$, and the time domain general solution of order ε can be obtained from Equation (23):

(26) $$\xi_{0m}=B_{0m}(T_{1},T_{2})e^{i\omega_{m}T_{0}}+{\bar{B}}_{0m}(T_{1},T_{2})e^{-i\omega_{m}T_{0}}$$

Among these, *B*_0*m*_ represents the amplitude of the *m*th mode, the subscript *m* represents the *m*th mode, and the subscript 0 represents the time scale under the severe change in *T*_0_ time. Substituting Equation (26) into the term composed of the order of $\varepsilon^{2}$ (Equation (24)),

(27) $${\ddot{\xi }}_{1m}(t)+\omega_{m}{}^{2}\xi_{1m}(t)=-2i\omega_{m}(\frac{\partial B}{\partial T_{1}}e^{i\omega_{m}T_{0}}-\frac{\partial \bar{B}}{\partial T_{1}}e^{-i\omega_{m}T_{0}})+\tilde{A}Q_{0}Z(\frac{e^{i2T_{0}}+e^{-i2T_{0}}}{2})(Be^{i\omega_{m}T_{0}}+\bar{B}e^{-i\omega_{m}T_{0}})$$

where $Q_{0}=\frac{\int_{0}^{1}\phi_{m}^{\prime \prime }\phi_{m}}{\int_{0}^{l}\phi_{m}^{2}dx}$. For the case of the first mode (*m* = 1), and considering the dimensionless frequency $\omega_{m}\approx 1$, the secular terms on the right side of Equation (27) are collected, and their sum made to be equal to 0; it is assumed that, when 1 = $\omega_{m}+\varepsilon \sigma$ and $\varepsilon T_{0}=T_{1}$, where σ is the tuned frequency, a solvability condition can be obtained. In addition, $\xi_{1m}$ can be obtained as follows:

(28) $$\xi_{1m}=\frac{-\tilde{A}Q_{0}ZB}{8(1+\omega_{m})}e^{i(2+\omega_{m})T_{0}}$$

Similarly, the terms are analyzed in the order of $\varepsilon^{3}$, Equations (26) and (28) are substituted into Equation (25), and the secular terms are collected and equated to 0, resulting in the solvability condition being found as follows:

(29) $$\begin{array}{l} 4\omega_{m}{}^{2}\gamma^{2}=\varepsilon^{2}(2\omega_{m}\sigma +\frac{\tilde{A}Q_{0}Z}{2}-\frac{\varepsilon \sigma Q_{0}\tilde{A}Z}{2\omega_{m}}-\varepsilon \frac{{\tilde{A}}^{2}Q_{0}{}^{2}Z^{2}}{16\omega_{m}{}^{2}}-\varepsilon \frac{{\tilde{A}}^{2}Q_{0}{}^{2}Z^{2}}{16(\omega_{m}+1)})\\ \left(-2\omega_{m}\sigma +\frac{\tilde{A}Q_{0}Z}{2}-\frac{\varepsilon \sigma Q_{0}\tilde{A}Z}{2\omega_{m}}+\varepsilon \frac{{\tilde{A}}^{2}Q_{0}{}^{2}Z^{2}}{16\omega_{m}{}^{2}}+\varepsilon \frac{{\tilde{A}}^{2}Q_{0}{}^{2}Z^{2}}{16(\omega_{m}+1)}\right) \end{array}$$

If γ is a positive number, the system will be unstable; in cases where γ>0 is set, then the tuned frequency of the unstable region can be determined from Equation (29), and the inequality can be expressed as the following equations:

(30) $$-\frac{\tilde{A}Q_{0}Z}{2}+\varepsilon \frac{{\tilde{A}}^{2}Q_{0}{}^{2}Z^{2}}{16\omega_{m}{}^{2}}+\varepsilon \frac{{\tilde{A}}^{2}Q_{0}{}^{2}Z^{2}}{16(\omega_{m}+1)}<2\omega_{m}\sigma -\frac{\varepsilon \sigma Q_{0}\tilde{A}Z}{2\omega_{m}}$$

(31) $$\frac{\tilde{A}Q_{0}Z}{2}+\varepsilon \frac{{\tilde{A}}^{2}Q_{0}{}^{2}Z^{2}}{16\omega_{m}{}^{2}}+\varepsilon \frac{{\tilde{A}}^{2}Q_{0}{}^{2}Z^{2}}{16(\omega_{m}+1)}>2\omega_{m}\sigma +\frac{\varepsilon \sigma Q_{0}\tilde{A}Z}{2\omega_{m}}$$

For the case of the second mode (*m* = 2), and considering the dimensionless frequency $\omega_{m}\approx 2$, consistent with the above procedure, the inequality can be obtained as follows:

(32) $$16\omega_{m}{}^{4}\mu^{2}-16\omega_{m}{}^{2}(\mu^{2}\omega_{m}{}^{2}+4\omega_{m}{}^{2}\sigma^{2}+4\omega_{m}\sigma \frac{{\tilde{A}}^{2}Q_{0}{}^{2}Z^{2}}{8(\omega_{m}{}^{2}-1)}-{\left(\frac{{\tilde{A}}^{2}Q_{2}{}^{2}Z^{2}}{16(\omega_{m}+1)}\right)}^{2}+{\left(\frac{{\tilde{A}}^{2}Q_{0}{}^{2}Z^{2}}{8(\omega_{m}{}^{2}-1)}\right)}^{2})>16\omega_{m}{}^{4}\mu^{4}$$

After solving Equation (32), the σ can be obtained as follows:

(33) $$\sigma =\frac{-4\omega_{m}\frac{{\tilde{A}}^{2}Q_{0}{}^{2}Z^{2}}{8(\omega_{m}{}^{2}-1)}}{8\omega_{m}{}^{2}}\pm \frac{\sqrt{16\omega_{m}{}^{2}{\left(\frac{{\tilde{A}}^{2}Q_{0}{}^{2}Z^{2}}{8(\omega_{m}{}^{2}-1)}\right)}^{2}+16\omega_{m}{}^{2}(\omega_{{}^{{}_{m}}}{}^{2}\mu^{2}-{\left(\frac{{\tilde{A}}^{2}Q_{2}{}^{2}Z^{2}}{16(\omega_{m}+1)}\right)}^{2}+{\left(\frac{{\tilde{A}}^{2}Q_{0}{}^{2}Z^{2}}{8(\omega_{m}{}^{2}-1)}\right)}^{2})}}{8\omega_{m}{}^{2}}$$

From Equations (30), (31) and (33), the unstable frequency regions of the system can be obtained. In the following Sections, the correctness of the unstable regions of the system will be further analyzed and verified.

### 3.2. Frequency Response

In this section, the frequency response of the system under parametric excitation will be analyzed. The orthogonality of the mode shape is utilized to decouple the dynamic equations at each scale. Introducing a simple harmonic disturbance external force *q_m_* to the system yields the following equations for each time scale:

Equation of order $\varepsilon^{1}$:

(34) $${\ddot{\xi }}_{0m}(t)+\omega_{m}{}^{2}\xi_{0m}(t)=0$$

Equation of order $\varepsilon^{2}$:

(35) $${\ddot{\xi }}_{1m}(t)+\omega_{m}{}^{2}\xi_{1m}(t)=-2\frac{\partial^{2}}{\partial T_{0}\partial T_{1}}\xi_{0m}(t)+\tilde{A}P(t)\frac{\int_{0}^{1}\phi_{m}^{\prime \prime }\phi_{m}}{\int_{0}^{l}\phi_{m}^{2}dx}\xi_{0m}$$

Equation of order $\varepsilon^{3}$:

(36) $$\begin{array}{l} {\ddot{\xi }}_{2m}(t)+\omega_{m}{}^{2}\xi_{2m}(t)=-2\frac{\partial^{2}}{\partial T_{0}\partial T_{1}}\xi_{1m}(t)-\frac{\partial^{2}}{\partial T_{1}{}^{2}}\xi_{0m}-2\frac{\partial^{2}}{\partial T_{0}\partial T_{2}}\xi_{0m}(t)-\mu \frac{\partial }{\partial T_{0}}\xi_{0m}+\tilde{A}P(t)\frac{\int_{0}^{1}\phi_{m}^{\prime \prime }\phi_{m}}{\int_{0}^{l}\phi_{m}^{2}dx}\xi_{1m}\\ +\frac{1\tilde{A}\int_{0}^{1}\phi_{m}^{\prime \prime }\phi_{m}}{2\int_{0}^{1}\phi_{m}{}^{2}}\xi_{0m}{}^{3}\int_{0}^{1}{\phi^{\prime }}_{m}^{2}dx+q_{m} \end{array}$$

In order to analyze the frequency response of the system, the frequency of the external force $\Omega =\omega_{m}+\varepsilon \sigma$ is introduced, where Ω is the frequency of the external force, and σ is the tuned frequency near the natural frequency of the *m*th mode $\omega_{m}$, so the external force can be expressed in the following form:

(37) $$q_{m}={\overset{⌢}{q}}_{m}e^{i\Omega T_{0}}={\overset{⌢}{q}}_{m}e^{i(\omega_{m}+\varepsilon \sigma )T_{0}}={\overset{⌢}{q}}_{m}e^{i\omega_{m}T_{0}}e^{i\sigma T_{0}}$$

The general solution of the time domain is expressed as follows:

(38) $$\xi_{j,m}=B_{j,m}(T_{1},T_{2})e^{-i\zeta_{m}}e^{i\omega_{m}T_{0}}+{\bar{B}}_{j,m}(T_{1},T_{2})e^{i\zeta_{m}}e^{-i\omega_{m}T_{0}}$$

where $\zeta_{m}$ is the phase angle, *B_m_* represents the amplitude of the *m*th mode, subscript *m* represents the *m*th mode, and subscript *j* represents different time scales. In the following, the first mode (*m* = 1) and $\omega_{m}\approx 1$ are taken as an example, and Equation (38) is substituted into Equation (35); the secular terms on the right hand side of Equation (35) are collected into the following equation:

(39) $${\ddot{\xi }}_{1m}(t)+\omega_{m}{}^{2}\xi_{1m}(t)=\frac{1}{2}\tilde{A}Q_{0}ZB_{m}e^{-i\zeta_{m}}e^{i(2+\omega_{m})T_{0}}$$

The expression $\xi_{1m}=\frac{-\tilde{A}Q_{0}ZB_{m}}{8(1+\omega_{m})}e^{-i\zeta_{m}}e^{i(2+\omega_{m})T_{0}}$ can then be obtained. Similarly, the $\xi_{1m}$ for the second mode (m=2, $\omega_{m}\approx 2$) can be obtained as $\xi_{1m}=\frac{-\tilde{A}Q_{0}ZB_{m}}{8(1+\omega_{m})}e^{-i\zeta_{m}}e^{i(2+\omega_{m})T_{0}}+\frac{\tilde{A}Q_{0}Z{\bar{B}}_{m}}{8(\omega_{m}-1)}e^{i\zeta_{m}}e^{i(2-\omega_{m})T_{0}}$.

After isolating the secular terms in Equations (34)–(36) and equating them to 0, the solvability condition for each mode can be derived. To construct the tuned frequency–response diagram (i.e., the fixed-point plot) of the dimensionless amplitude Bm for this nonlinear system, the NEQNF subroutine within IMSL, along with the Levenberg–Marquardt algorithm, is employed.

### 3.3. Time Response of Beam Amplitude

In this section, the correctness of the fixed-point plots and the initial voltage generation benefit of this model will be verified. The fourth-order Runge–Kutta (RK-4) method is used to obtain the time–response and phase plots. The piezoelectric equation and the elastic beam equation are solved simultaneously to obtain the theoretical voltage output, which is then used to verify the unstable region of the system.

The concept of small perturbation Is introduced, letting $\xi_{n}={\bar{\xi }}_{n}+{\tilde{\xi }}_{n}$, where ${\bar{\xi }}_{n}$ is the equilibrium term, ${\tilde{\xi }}_{n}$ is the perturbation term, and it is assumed that $W=({\bar{\xi }}_{n}+{\tilde{\xi }}_{n})\phi_{n}$. These assumptions are substituted into Equation (17), and the orthogonal method is then used to expand the electric beam equation as follows:

(40) $$\begin{array}{l} {\ddot{\tilde{\xi }}}_{n}{}^{}+{\tilde{\xi }}_{n}\frac{\int_{0}^{1}\phi_{m}\phi_{m}{}^{iv}dx}{\int_{0}^{1}\phi_{m}{}^{2}dx}+\mu {\dot{\tilde{\xi }}}_{n}\frac{\int_{0}^{1}\phi_{m}{}^{2}dx}{\int_{0}^{1}\phi_{m}{}^{2}dx}-\tilde{A}P(t){\tilde{\xi }}_{n}\frac{\int_{0}^{1}\phi_{m}\phi_{m}{}^{\prime \prime }dx}{\int_{0}^{1}\phi_{m}{}^{2}dx}\\ -\frac{1}{\int_{0}^{1}\phi_{m}{}^{2}dx}(\frac{\hat{k}\eta^{2}}{e^{Rt}}({\bar{\xi }}_{m}+{\tilde{\xi }}_{m})\int_{0}^{1}\phi_{m}(\int_{a}^{b}\phi_{m}^{\prime \prime }dx{)}^{2}(\int_{0}^{\tau }({\bar{\xi }}_{m}+{\tilde{\xi }}_{m}\dot{)}e^{Rt}dt)dx)=\frac{1}{2}\tilde{A}{\tilde{\xi }}^{3}\int_{0}^{1}\phi_{m}{{}^{\prime }}^{2}dx\frac{\int_{0}^{1}\phi_{m}\phi_{m}{}^{\prime \prime }dx}{\int_{0}^{1}\phi_{m}{}^{2}dx} \end{array}$$

The first mode ($m=1,\omega_{m}\approx 1$) and the second mode ($m=2,\omega_{m}\approx 2$) were substituted into Equation (40), and the phase plots of the piezoelectric beam system and time responses of the beam amplitudes were determined using the RK-4 method. The correctness of the fixed-point plots was verified using these results. At this point, the results from Equation (40) were also employed, and the theoretical output voltage was obtained by combining it with the voltage function (Equation (10)).

### 3.4. Parametric Excitation Analysis

**Figure 3:**
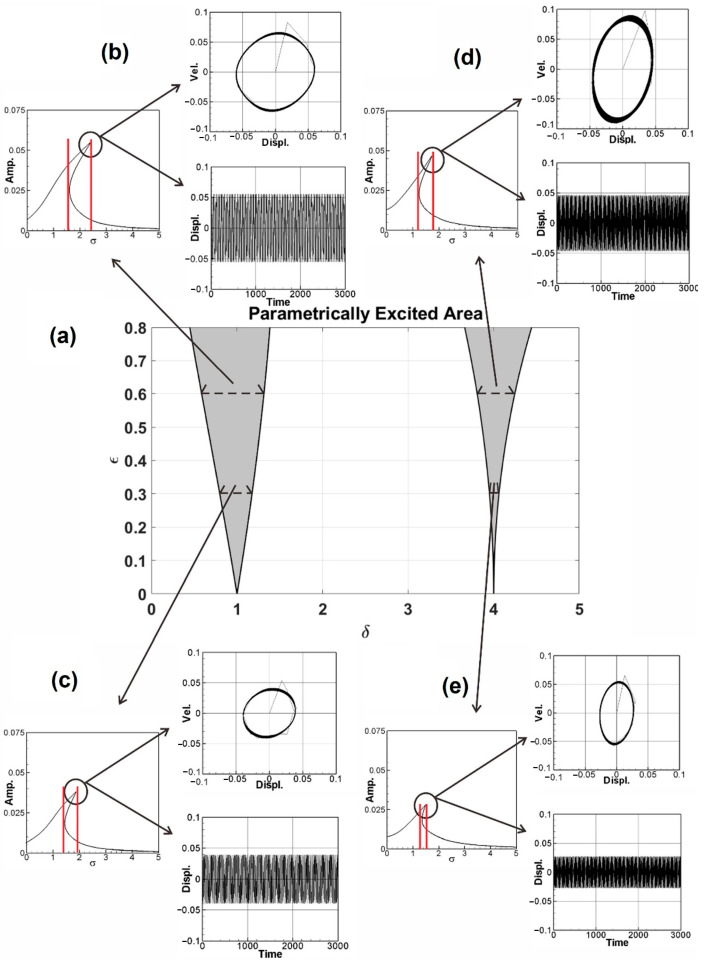
Verification of parametric excitation: (**a**) stable and unstable (shaded) regions in the parameter plane; (**b**–**e**) fixed-point plot, phase plot and time–response plot of the first mode and the second mode under different external forces.

The unstable region inequality equations of the parametric excitation system (Equations (30), (31) and (33)) were obtained in Section 3.1. In this section, the assumptions $1=\omega_{m}+\varepsilon \sigma ,\;1\approx \omega_{m}$, $2=\omega_{m}+\varepsilon^{2}\sigma ,\;2\approx \omega_{m}$, and $\delta =\omega_{m}{}^{2}$ are utilized. By substituting these assumptions into Equations (30), (31) and (33), respectively, the unstable region of the system can be determined, which is depicted as a gray area in Figure 3a. In this study, a shaker is connected to one end of the beam to simulate the magnitude and frequency of axial external forces. It can be seen from Figure 3a that when the values of the vertical axis (disturbance external forces) increase, the unstable region of the system also increases, and when the system is in the unstable region, a larger amplitude will be generated. In order to obtain better power generation efficiency in subsequent experiments, the frequency and amplitude of the actuator will be adjusted so that the system can obtain a larger amplitude to facilitate power conversion. The experimental verification of the phenomenon in Figure 3a will be discussed in the next section.

After determining the instability region of the system, numerical methods are used to verify its correctness. Figure 3a presents the unstable region of the system, and Figure 3b–e present the fixed-point plots of the system. The bend curves in the figures (in the range marked by the red lines) are jump phenomena. In the range of system frequencies, the system has a greater amplitude due to energy conversion, and the fixed-point plots are used to verify the correctness of the system parameter excitation instability region. The correctness of the instability region of the first mode of the system is first verified. Figure 3b,c present the fixed-point plots when the external force is equal to 0.6 and the external force is equal to 0.3 when the system frequency is equal to 1 ($\delta =\omega_{m}{}^{2}=1$). It can be seen from Figure 3b,c that the offset ratio of the fixed-point plots peaks is about 1.9335. Figure 3d,e present the fixed-point plots when the external force is equal to 0.6 and the external force is equal to 0.3 when the system frequency is equal to 2 ($\delta =\omega_{m}{}^{2}=4$). It can also be seen that the offset ratio of the fixed-point plots peaks in Figure 3d,e is about 4.0192. In Figure 3a, the ratios of the unstable regions in the external force equal to 0.6 and external force equal to 0.3 are 1.9332 and 4.0183, which is consistent with the ratios presented in the fixed-point plots in the above four cases.

Additionally, time–response plots and phase plots are created using the fourth-order Runge–Kutta method to verify the maximum amplitude point (indicated by a black circle) of fixed-point plots. Taking Figure 3b as an example, in the upper right corner is the phase plot. It can be seen that the system converges to multiple irregular shapes, which demonstrates the phenomenon of instability. In the lower right corner of Figure 3b is the time–response plot. It can be seen that the amplitude is consistent with the amplitude of the fixed-point plots, so the correctness of the fixed-point plots and the unstable region of the disturbance in the system parameters is verified.

### 3.5. Voltage Generation

**Figure 4:**
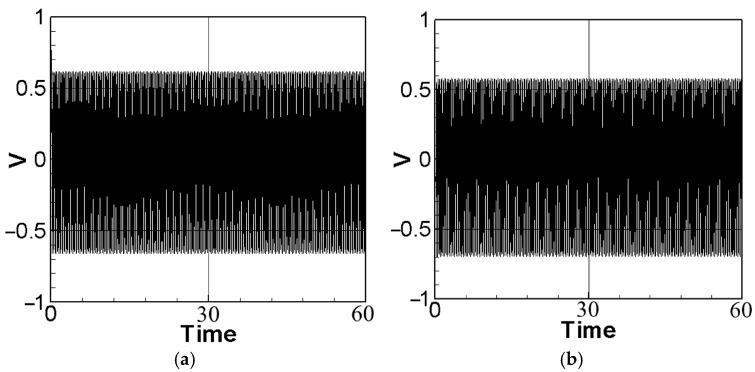

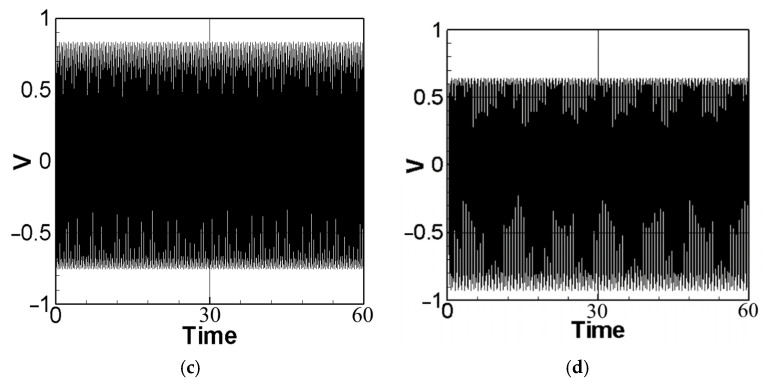
Theoretical voltage of each mode: (**a**) the first mode with PZT placed at the maximum amplitude position; (**b**) the first mode with PZT placed at the root; (**c**) the second mode with PZT placed at the maximum amplitude position; (**d**) the second mode with PZT placed at the root.

To achieve the best voltage generation efficiency, precise placement (repositioning) of the PZT at the maximum deformation (amplitude) location of the elastic beam is essential. The maximum amplitude can be determined by means of fixed-point plots. By combining the piezoelectric coupling nonlinear beam equation (Equation (40)) with the voltage function (Equation (10)) and setting the magnitude of the external force (actuator amplitude) to 0.6, The theoretical voltage output can be calculated using the fourth-order Runge–Kutta (RK-4) method. The results are presented as response graphs of voltage versus time, as depicted in Figure 4. Specifically, Figure 4a,b illustrate the theoretical voltage diagrams of the first mode when the PZT is placed at the maximum amplitude of the beam and at the root of the beam, respectively. Likewise, Figure 4c,d depict the theoretical voltage diagrams of the second mode with the PZT placed at the maximum amplitude and the root of the beam, respectively. Finally, the root mean square values are tabulated in Table 1 for easy comparison and analysis.

Based on the observations from Figure 4 and Table 1, it is evident that voltage generation increases with increasing system frequency, with higher frequency resulting in better voltage generation efficiency. Whether considering the first mode or the second mode of the beam, voltage generation at the maximum amplitude of the beam outperforms that at the root. Repositioning the piezoelectric patch to the middle of the elastic steel sheet enhances vibration energy harvesting. In this study, the patch is strategically placed at the point of maximum deformation during vibration, leading to an increase in the mechanical stress on the piezoelectric material. This heightened stress results in improved energy conversion efficiency and higher voltage generation. In the following sections, a comprehensive analysis will be undertaken to explore the benefits of voltage generation by introducing baffles at the maximum amplitude position of the beam to enhance the slapping force.

## 4. Voltage Generation Benefit Analysis of Slapping Force

**Figure 5:**
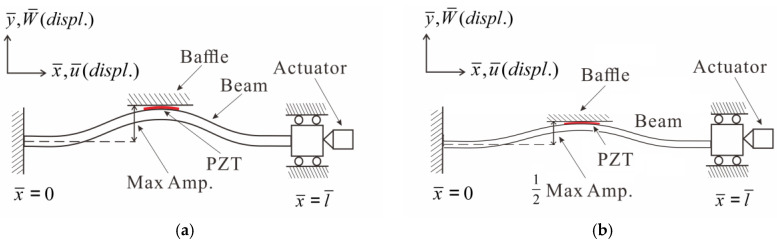
Theoretical framework diagram of slapping force with different amplitudes: (**a**) max. amp.; (**b**) 1/2 max. amp.

In this section, the findings of the theoretical model are presented. The installation of a piezoelectric patch at the point of maximum deformation, coupled with the addition of a baffle to introduce a slapping force, was investigated. The voltage generation efficiency of the baffle was compared at two key location: the maximum amplitude position of the elastic beam and the half-maximum amplitude position. For a clearer understanding, the detailed coordinate definitions and the components of the theoretical model are illustrated in Figure 5a,b, respectively.

The impact force of the elastic beam hitting the baffle is regarded as the slapping force on the PZT, and the slapping force is expressed as:

(41) $$\bar{F}=\rho A{\ddot{\bar{W}}}_{F}\bar{\delta }(\bar{t}-\bar{T}),\;\bar{t}>0$$

$\bar{T}$ is the slapping period, $\bar{\delta }(\bar{t})$ is the Dirac function.

Divide Equation (41) by $\frac{EI}{\rho A{\bar{l}}^{3}}$ to obtain the dimensionless slapping force, $\bar{F}={\ddot{W}}_{F}{}^{}\bar{\delta }(t-T)$. Then, add Equation (41) to Equation (12) to obtain the dimensionless nonlinear beam equation with the slapping force as follows:

(42) $$\ddot{W}+W^{iv}+\mu \dot{W}-\tilde{A}P_{\left(t\right)}W^{\prime \prime }-\eta^{2}(\int_{a}^{b}W^{\prime \prime }dx)v=\frac{1}{2}\tilde{A}[\int_{0}^{1}{W^{\prime }}^{2}dx]W^{\prime \prime }+{\ddot{W}}_{F}{}^{}\bar{\delta }(t-T)$$

Using the small perturbation method, and letting $\xi_{n}={\bar{\xi }}_{n}+{\tilde{\xi }}_{n}$, where ${\bar{\xi }}_{n}$ is the equilibrium term and ${\tilde{\xi }}_{n}$ is the perturbation term, assuming that $W=({\bar{\xi }}_{n}+{\tilde{\xi }}_{n})\phi_{n}$ and substituted into Equation (42), the beam equation with slapping force is expanded using the orthogonal method as follows:

(43) $$\begin{array}{l} {\ddot{\tilde{\xi }}}_{n}{}^{}+{\tilde{\xi }}_{n}\frac{\int_{0}^{1}\phi_{m}\phi_{m}{}^{iv}dx}{\int_{0}^{1}\phi_{m}{}^{2}dx}+\mu {\dot{\tilde{\xi }}}_{n}{}^{}\frac{\int_{0}^{1}\phi_{m}{}^{2}dx}{\int_{0}^{1}\phi_{m}{}^{2}dx}-\tilde{A}P_{\left(t\right)}{\tilde{\xi }}_{n}\frac{\int_{0}^{1}\phi_{m}\phi_{m}{}^{\prime \prime }dx}{\int_{0}^{1}\phi_{m}{}^{2}dx}\\ -\frac{1}{\int_{0}^{1}\phi_{m}{}^{2}dx}(\frac{\hat{k}\eta^{2}}{e^{Rt}}({\bar{\xi }}_{m}+{\tilde{\xi }}_{m})\int_{0}^{1}\phi_{m}(\int_{a}^{b}\phi_{m}^{\prime \prime }dx{)}^{2}(\int_{0}^{t}({\bar{\xi }}_{m}+{\tilde{\xi }}_{m}\dot{)}e^{Rt}dt)dx)\\ =\frac{1}{2}\tilde{A}{\tilde{\xi }}^{3}\int_{0}^{1}\phi_{m}{{}^{\prime }}^{2}dx\frac{\int_{0}^{1}\phi_{m}\phi_{m}{}^{\prime \prime }dx}{\int_{0}^{1}\phi_{m}{}^{2}dx}A+\frac{\int_{a}^{b}\phi_{m}{}^{2}dx}{\int_{0}^{1}\phi_{m}{}^{2}dx}({\bar{\xi }}_{m}+{\tilde{\xi }}_{m}\ddot{)}\bar{\delta } \end{array}$$

**Figure 6:**
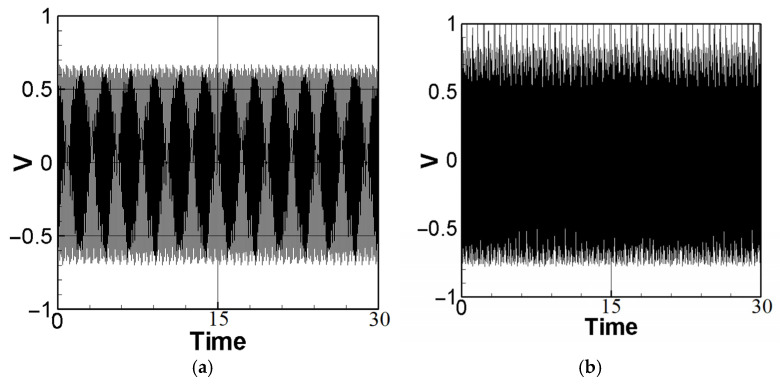

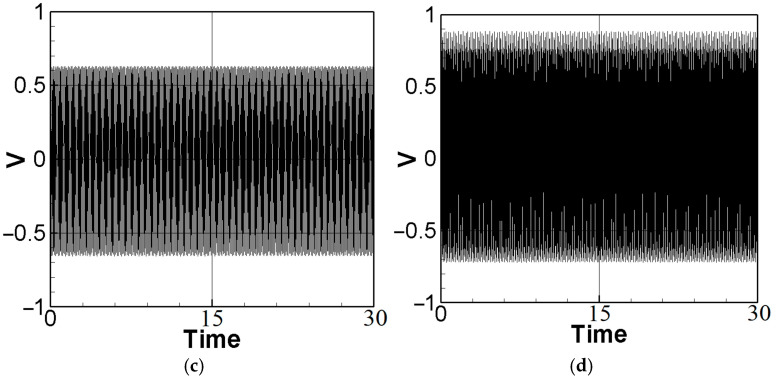
Dimensionless theoretical voltage diagram of slapping force with different amplitudes: (**a**) first mode, Max. Amp; (**b**) second mode, max. amp. (**c**) first mode, 1/2 max. amp. (**d**) second mode, 1/2 max. amp.

Substitute the first mode ($m=1,\omega_{m}\approx 1$) and the second mode ($m=2,\omega_{m}\approx 2$) into Equation (43), and use the fourth-order Runge–Kutta (RK-4) method to calculate the theoretical voltage value. The magnitude of the external force was fixed at 0.6, and the theoretical voltage value of the additional slapping force was calculated using the RK-4 numerical method, as depicted in Figure 6a–d. The root mean square values were compiled and are presented in Table 2.

It can be seen from Figure 6a–d and Table 2 that voltage generation will increase with increasing system frequency, resulting in higher voltage generation efficiency. In addition, due to the higher impacting frequency of the second mode, its electric power generation efficiency is also higher than that of the first mode. According to the numerical analysis results (Table 1 and Table 2), it can be seen that the additional slapping force has higher voltage generation efficiency, and the voltage generation efficiency generated by the higher deformation and slapping force at the maximum amplitude is the best.

The methodology used in this study takes an innovative approach by combining the principles of piezoelectric energy conversion and mechanical augmentation. Rather than solely focusing on the traditional placement of piezoelectric materials, the strategic positioning of the patch for heightened energy conversion is explored. Furthermore, the incorporation of the baffle introduces an inventive dimension to energy harvesting, resulting in a remarkable enhancement of voltage generation. This unique methodological framework contributes to the novelty of this study, as it goes beyond the established paradigms of vibration energy harvesting.

## 5. Experimental Analysis

**Figure 7:**
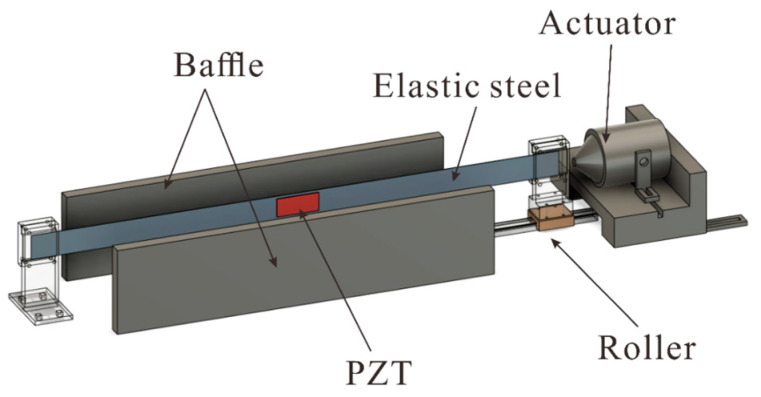
Schematic diagram of the slapping energy harvesting system for axial excitation.

In order to further verify the results of this study, a simple experiment was set up, as shown in Figure 7. This energy harvesting system was divided into three parts, namely, the actuator, the elastic steel sheet installed with the piezoelectric patch, and the baffle for generating slapping force. The design principle was to use the disturbance generated by the actuator at the end of the elastic steel to cause buckling vibration in the steel sheet equipped with a piezoelectric patch, and to collect the vibration energy generated by the deformation and slapping of the steel.

### 5.1. Experimental Setup Design

**Figure 8:**
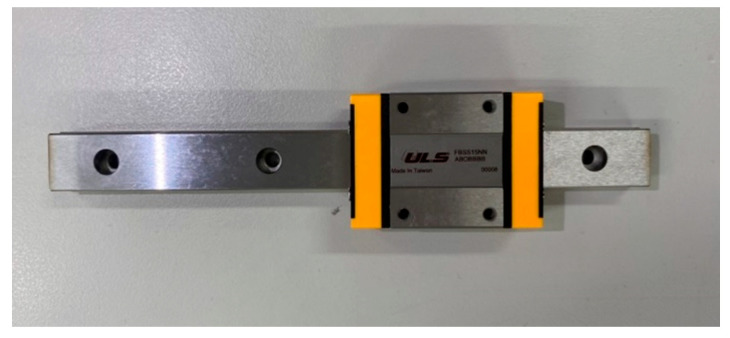
Slide rails.

**Figure 9:**
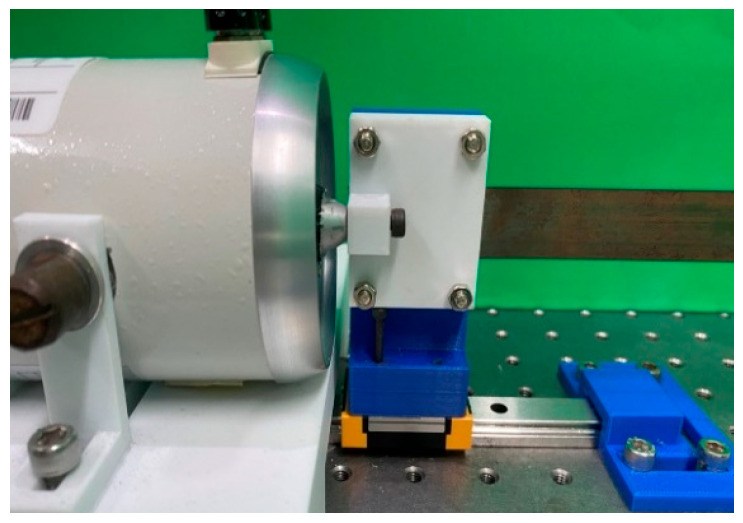
Actuator connected to the moveable endpoints.

**Figure 10:**
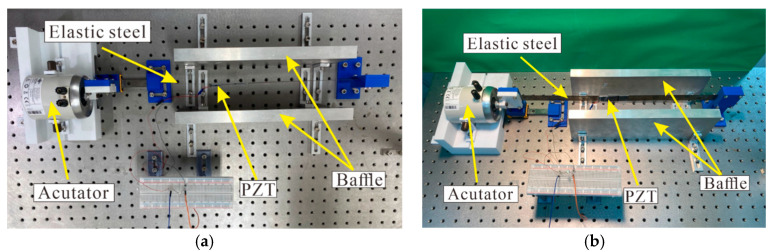
Experimental setup: (**a**) top view; (**b**) oblique view.

In order to confirm whether this device works, the VEH system’s parts were made using a 3D printer. One end of the steel was fixed, and the other end was provided with a horizontally movable slide rail (Figure 8). Then, the actuator was used to excite the system. The end point of the steel was fixed to the actuator with screws (Figure 9) to ensure that the frequency of the actuator excitation was able to directly affect the frequency of the elastic steel. In this way, by changing the frequency of the actuator, the unstable region of the system could be excited, and the piezoelectric patch could be placed at the maximum amplitude position of the steel to harvest energy. Additional baffles were added at the location of maximum vibration amplitude to generate slapping force to increase voltage generation efficiency. The experimental setup is shown in Figure 10.

### 5.2. Natural Frequency and Internal Resistance

**Figure 11:**
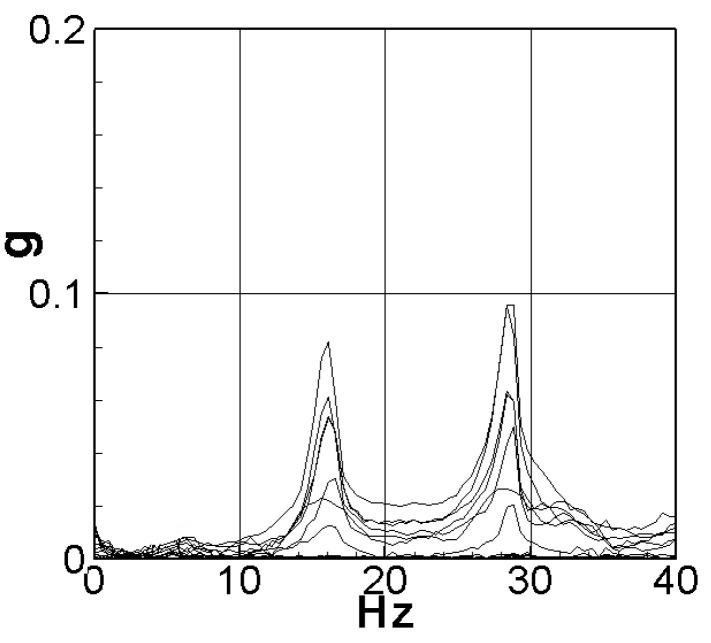
Natural frequency of the elastic steel sheet.

Before commencing the experiment, the natural frequency of the elastic steel was verified using an impact hammer, accelerometer, and fast Fourier transform (from the imc© system, TÜV Rheinland, Kölle, Germany). These tools allowed us to determine the first two natural frequencies of the steel, which were found to be 16.2 Hz and 28.8 Hz, as illustrated in Figure 11. To analyze the voltage generation of the slapping force energy harvesting system, it is essential to introduce resistance into the system in the form of the system load. By incorporating an appropriate additional resistance value that is close to the internal resistance of the system, the optimal electrical power output can be achieved, enabling us to maximize the system’s overall power efficiency. The following describes the estimation of internal resistance. First, the electric power equation is as follows:

(44) $$P=\bar{I}\bar{V}={\bar{I}}^{2}R=\frac{{\bar{V}}^{2}}{R}$$

where *P* is power, $\bar{I}$ denotes current, $\bar{V}$ represents voltage, and *R* is resistance. Thevenin’s theorem was used to find the internal resistance. Thevenin’s theorem gives

(45) $${\bar{V}}_{L}=\frac{R_{L}}{R_{T}+R_{L}}{\bar{V}}_{T}$$

where $R_{T}$ is internal resistance, $R_{L}$ denotes the loading resistance, ${\bar{V}}_{T}$ is the open circuit voltage, and ${\bar{V}}_{L}$ is the loading voltage. From Equations (44) and (45):

(46) $$P=\frac{{\bar{V}}_{L}^{2}}{R_{L}}=\frac{\frac{R_{L}^{2}}{{\left(R_{T}+R_{L}\right)}^{2}}{\bar{V}}_{T}^{2}}{R_{L}}=\frac{R_{L}{\bar{V}}_{T}^{2}}{{\left(R_{T}-R_{L}\right)}^{2}+4R_{T}R_{L}}=\frac{{\bar{V}}_{T}^{2}}{\frac{{\left(R_{T}-R_{L}\right)}^{2}}{R_{L}}+4R_{T}}$$

**Figure 12:**
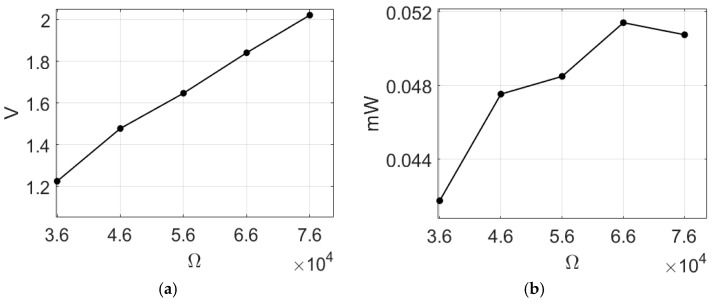
Voltages and powers for different load resistances: (**a**) ohm–volt diagram; (**b**) ohm–power diagram.

The optimal electric power output is achieved when the loading resistance matches the internal resistance of the system. To determine the internal resistance of this system, a 20 K ohm load was arbitrarily selected for the system, and an output voltage of 0.82865 volts was measured. By substituting this result into Equations (45) and (46), the theoretical internal resistance value was calculated to be 65.9 K ohms. In a subsequent step, the load resistance within the range of 36 K to 76 K ohms was experimentally tested, and the corresponding output voltage and power were measured, as presented in Table 3. The imc© system was utilized to obtain the open circuit voltage of the system, which was then processed through the Butterworth filter to acquire the voltage measurements. Specifically, the first mode of this system was excited without any baffles, resulting in an average open-circuit voltage of 3.625 volts (as indicated in Table 3). Based on the data presented in Table 3, the ohm–volt diagram and the ohm–power diagram were constructed, and are depicted in Figure 12a,b, respectively. These diagrams reveal that the highest output power of 0.0514 mW was attained when the load resistance was set to 66 K ohms. Therefore, in the subsequent sections, when evaluating the power generation efficiency of the system, a load resistance of 66 K ohms was utilized as the experimental configuration.

### 5.3. System Voltage Measurement

**Figure 13:**
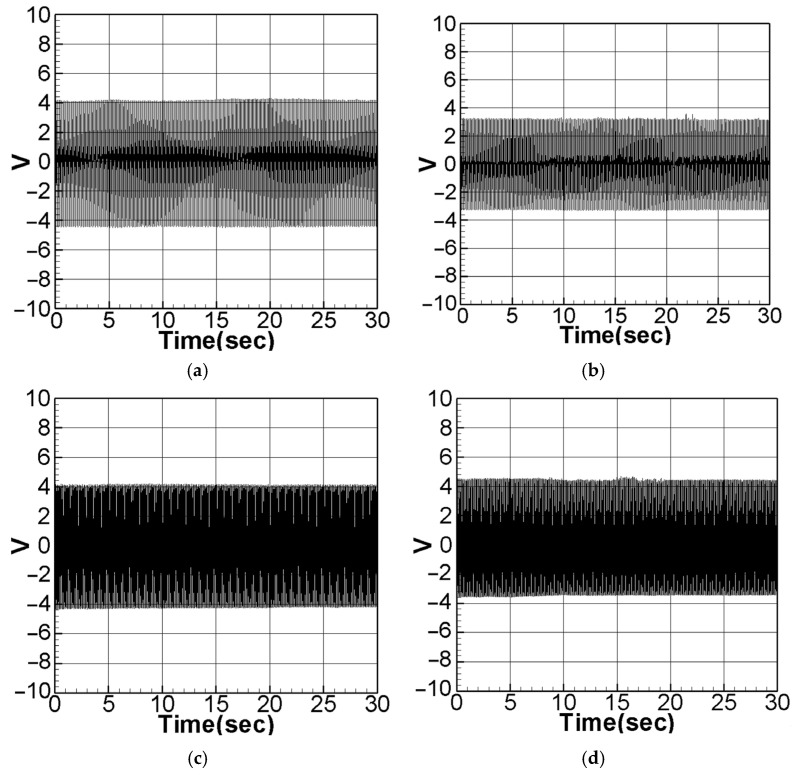
Experimental voltage diagram without slapping force: (**a**) the first mode with PZT placed at the maximum amplitude position of the elastic steel; (**b**) the first mode with PZT placed at the root of the elastic steel; (**c**) the second mode with PZT placed at the maximum amplitude position of the elastic steel; (**d**) the second mode with PZT placed at the root of the elastic steel.

Based on the measured natural frequencies, the first mode was excited at 16.2 Hz in this experiment, while the second mode will be excited at 28.8 Hz. Measurements were initially conducted on the system without any additional slapping force. The voltage generation efficiency of the PZT was measured when installed at both the root and at the maximum amplitude position of the elastic steel. The voltage output of the system was recorded, and subsequently, the root mean square value was calculated from the collected data. Next, a baffle was introduced at the maximum amplitude position of the elastic steel, and the voltage generation efficiency was measured when the slapping force was applied at both the maximum amplitude position and at the half-maximum amplitude position of the steel. In this part of the experiment, the first mode was excited at 16.2 Hz and the second mode at 28.8 Hz. To calculate the output voltage of the system, a 66 K load resistance was connected in series. The voltage of the non-slapping force system was then measured, and the voltage generation efficiency of the elastic beam was measured when the PZT was installed at the maximum amplitude and at the root, respectively (Figure 13a–d). The results were compiled and are presented as root mean square values in Table 4.

**Figure 14:**
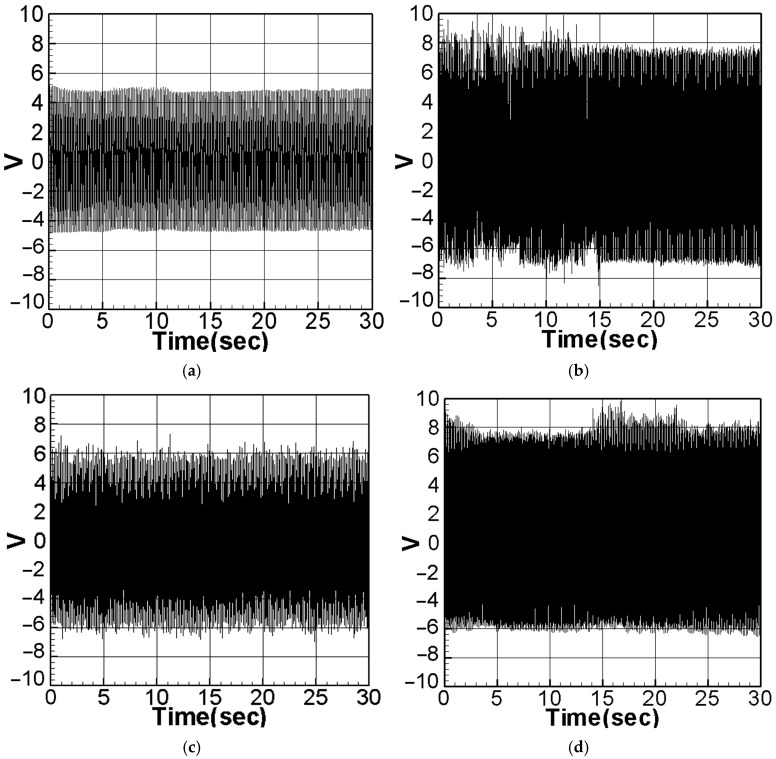
Experimental voltage output with the slapping force: (**a**) the first mode, baffle placed at the maximum amplitude position of the elastic steel; (**b**) the second mode, baffle placed at the maximum amplitude position of the elastic steel; (**c**) the first mode, baffle placed at the half-maximum amplitude location of the elastic steel; (**d**) the second mode, baffle placed at the half-maximum amplitude location of the elastic steel.

From the experimental results, the voltage generation efficiency generated at the maximum amplitude position can be observed to be better in the condition with no slapping force. Subsequently, a baffle was installed in this system to analyze the effect of the slapping force. The voltage generation efficiency was measured of the baffle located at the maximum amplitude position of the elastic beam and at the half-maximum amplitude position, respectively (Figure 14a–d). The root mean square results are presented Table 5. From the above voltage measurement results of the slapping force, it can be seen that the voltage generation efficiency at the maximum amplitude is also better. In the next Section, the numerical analysis results will be compared with the experimental results.

## 6. Verification of Experimental Results with Theory

**Figure 15:**
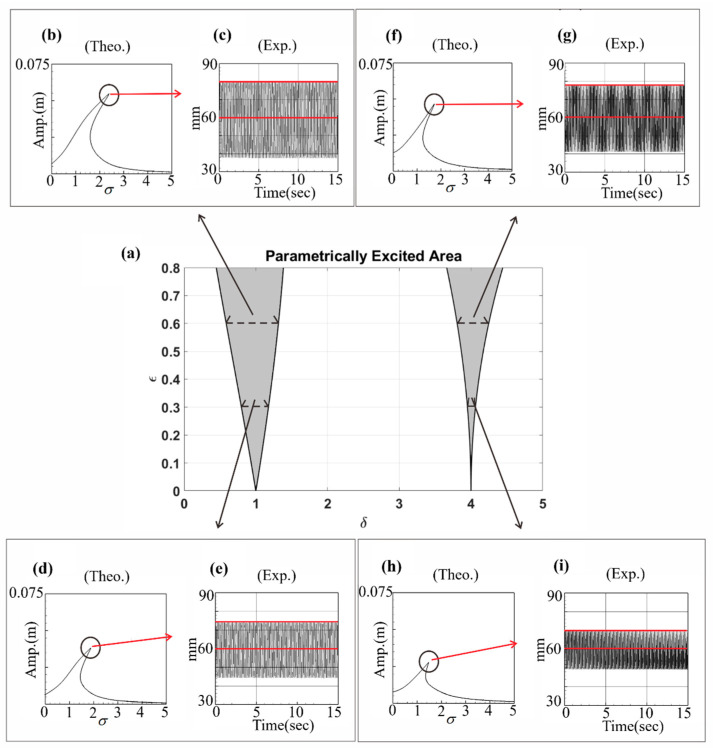
Experimental verification of the maximum amplitudes of the fixed-point plots. (**a**) the unstable regions of the parametric excitation; (**b**) the fixed points plot of dimensionless frequency = 1, excitation force = 0.6; (**c**) experimental measured displacement, red lines represent the range of the amplitude of case (**b**); (**d**) the fixed points plot of dimensionless frequency = 0.1, excitation force = 0.3; (**e**) experimental measured displacement, red lines represent the range of the amplitude of case (**d**); (**f**) the fixed points plot of dimensionless frequency = 4, excitation force = 0.6; (**g**) experimental measured displacement, red lines represent the range of the amplitude of case (**f**); (**h**) the fixed points plot of dimensionless frequency = 4, excitation force = 0.3; (**i**) experimental measured displacement, red lines represent the range of the amplitude of case (**h**).

Figure 15 presents the experimental verification of the maximum amplitude. For instance, in Figure 15c, a laser displacement gauge was utilized to measure the amplitude of the buckling in the elastic steel sheet. The measured vibration values ranged between 82 mm and 38 mm, and the maximum vibration amplitude was determined to be 2.2 cm, after dividing the value by 2. Considering that the length of the elastic steel sheet is 42 cm, the experimental maximum amplitude after dimensionless conversion was found to be 0.0523. Comparing this experimental result with the theoretical maximum amplitude of 0.0547, calculated using fixed-point plots in Figure 15b, the error was calculated to be 4.7%. This validates the accuracy of the theory regarding the maximum amplitude point of the fixed-point plots.

**Figure 16:**
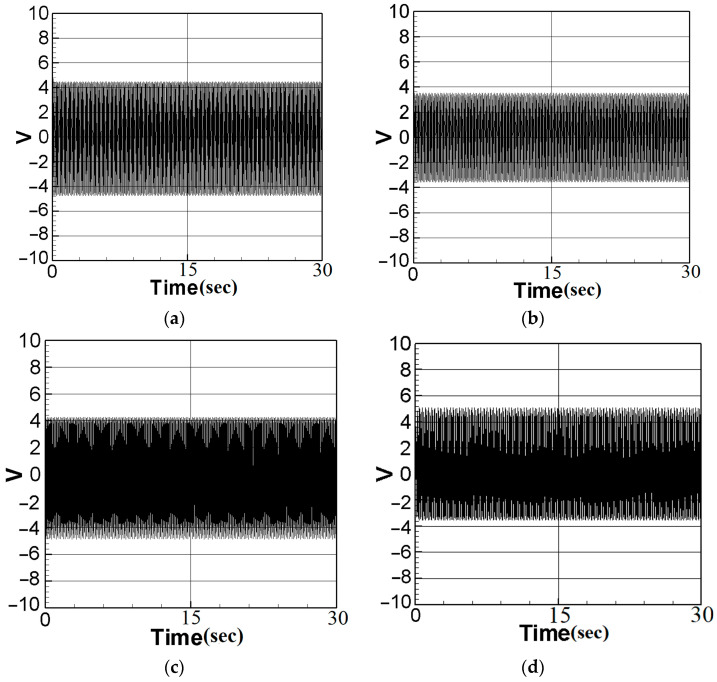
Dimensional theoretical voltage diagram without slapping force: (**a**) the first mode with PZT placed at the maximum amplitude position of the elastic steel; (**b**) the first mode with PZT placed at the root of the elastic steel; (**c**) the second mode with PZT placed at the maximum amplitude position of the elastic steel; (**d**) the second mode with PZT placed at the root of the elastic steel.

Next, the dimensional theoretical voltage diagrams of the non-slapping force were computed using the RK-4 method (Figure 16a–d). The theoretical and experimental results are then summarized in Table 6. Experimental and theoretical verification also confirmed that the best voltage output effect was achieved by placing PZT in the middle of the elastic steel. With these considerations taken into account, theoretical values were derived, and the dimensional voltage values were calculated. Subsequently, a comparison between these theoretical values and the experimental measurements was conducted to assess their correlation and agreement.

As observed from Table 6, the errors between the theoretical results (with dimension) and the experimental voltage values do not exceed 10%, providing sufficient evidence to validate the accuracy of the theory in the slap-free system.

**Figure 17:**
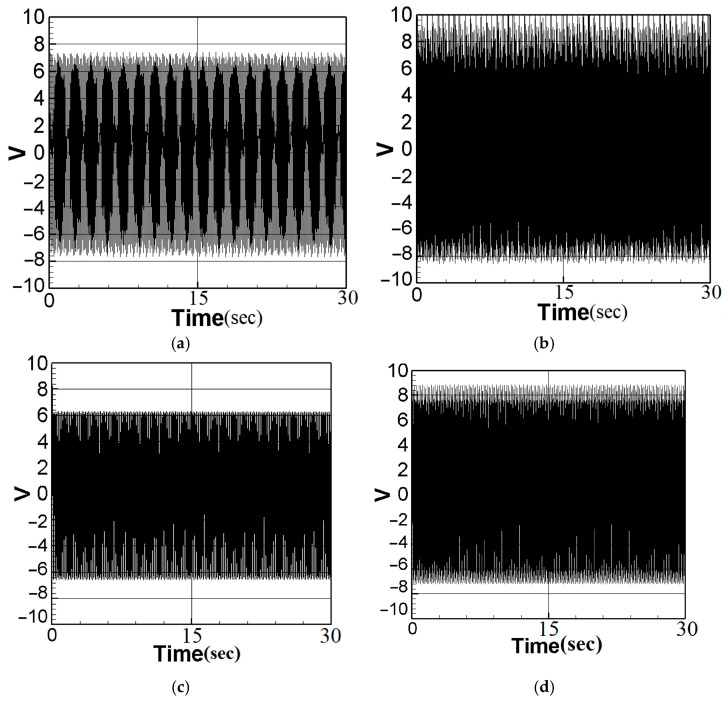
Theoretical dimensional voltage output with the slapping force: (**a**) the first mode, baffle placed at the maximum amplitude position of the elastic steel; (**b**) the second mode, baffle placed at the maximum amplitude position of the elastic steel; (**c**) the first mode, baffle placed at the half-maximum amplitude position of the elastic steel; (**d**) the second mode, baffle placed at the half-maximum amplitude position of the elastic steel.

Subsequently, the dimensional theoretical voltage diagram was calculated, considering the slapping force, using numerical methods (Figure 17a–d). The results, along with the corresponding experimental data, are presented in Table 7. Furthermore, the experimental voltage values from the systems both without slapping force and with slapping force were compiled and are presented for direct comparison in Table 8.

As shown in Table 7, the errors between the theoretical predictions and the experimental voltage values do not exceed 10%, providing substantial evidence to validate the accuracy of the theory concerning the slapping force system. Moreover, Table 8 clearly demonstrates that the voltage generation benefits of additional slapping force are significantly higher than those without slapping force. According to the experimental results, the voltage value for the first mode without slapping force is approximately 1.9096 V. However, after considering the effect of the slap, the voltage increases to around 2.7412 V. Similarly, for the second mode, the voltage increases from approximately 2.7845 V to about 3.8263 V. These findings confirm that the combined action of deformation and slapping force does indeed lead to higher power generation efficiency.

**Figure 18:**
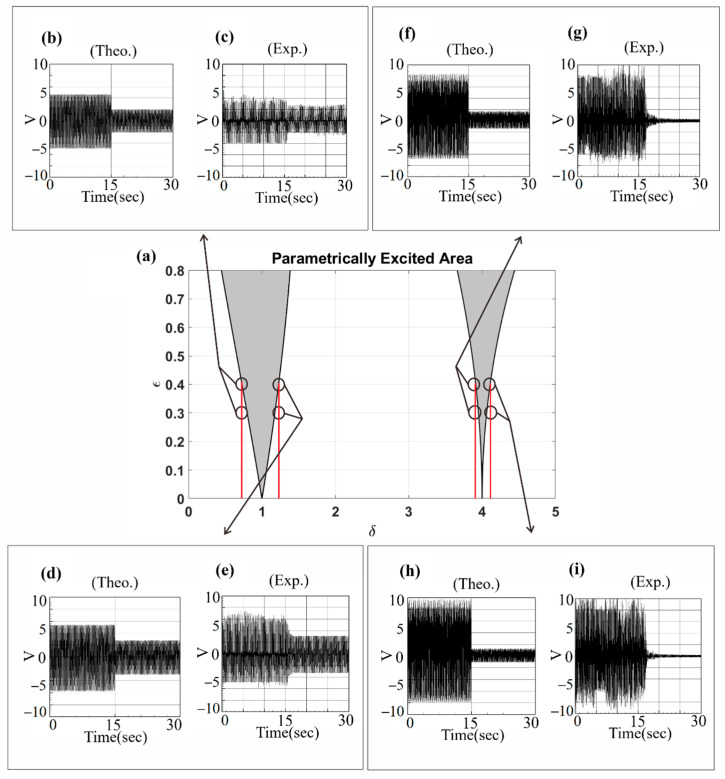
Verification of voltage generation efficiency of the parametric excitation VEH system: (**a**) unstable region of parametric excitation VEH system, the red lines represent the range of unstable region frequencies; (**b**–**i**) theoretical and experimental voltage diagrams of different external forces.

**Figure 19:**
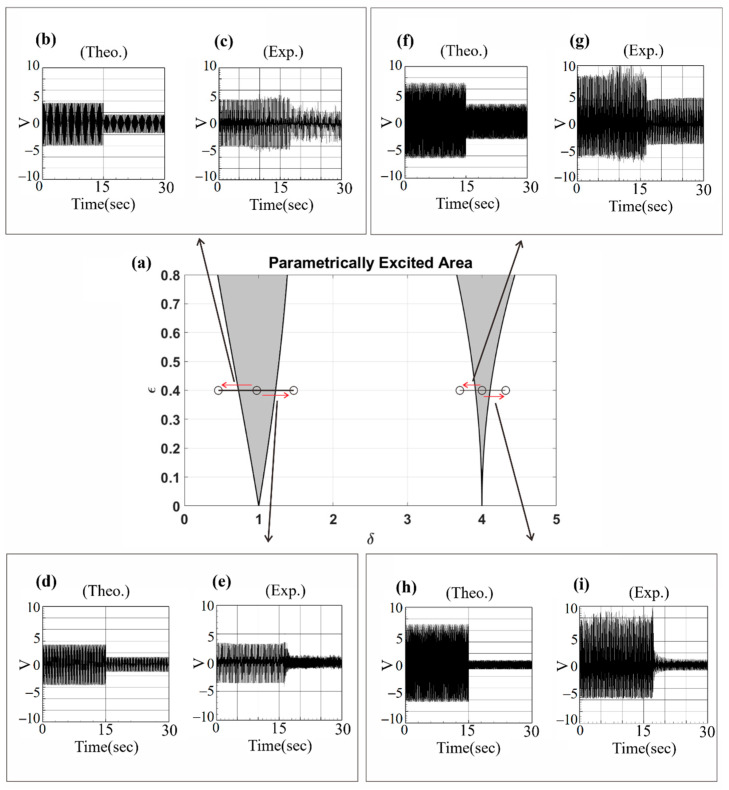
Verification of voltage generation efficiency of the parametric excitation VEH system: (**a**) unstable region of parametric excitation VEH system, red arrows represent the directions of frequency changes; (**b**–**i**) theoretical and experimental voltage diagrams for different input frequencies.

To further investigate the voltage generation efficiency in the unstable region depicted in Figure 3a, the RK-4 numerical method was employed to calculate the dimensional theoretical voltage of the system in both the unstable and stable regions. The results were then compared with the experimental data, as illustrated in Figure 18. The output voltage values are also presented in Table 9 for detailed analysis. In the unstable region of the system, the amplitude becomes larger, resulting in higher power generation efficiency. In Figure 18, the results of experiments conducted with fixed system frequency while adjusting the external force to assess system stability are presented. For instance, in Figure 18c, the measurements are presented for when the system frequency was set to 13.8 Hz and the external force to 0.4, causing the system to enter the unstable region. Measurements were taken for 15 s in this state. Subsequently, the external force was adjusted to 0.3, bringing the system into a stable region, and measurements were continued for another 15 s. The experimental results clearly demonstrated a significant voltage drop in the stable region, consistent with the theoretical prediction shown in Figure 19b. This observation confirmed the relationship between system stability and voltage generation efficiency within the scope of this study.

From the results presented in Table 9, it is evident that, irrespective of the fixed system frequency, a higher voltage generation efficiency is observed when the external force is set at 0.4, corresponding to the unstable region. Conversely, lowering the external force magnitude to 0.3, within the stable region, significantly reduces the voltage generation benefit. The error between theoretical calculations and experimental measurements remains below 10%, confirming the accuracy of predicting unstable range of the parameter disturbance. Moreover, to explore the system’s stability, the external force remained constant at 0.4, while the system frequency was varied in order to observe its behavior. The voltage generation efficiency within both stable and unstable regions was assessed. Using the RK-4 numerical method, the dimensional theoretical voltages of the system were computed for both regions. Subsequently, these theoretical values were compared and validated against experimental results, as depicted in Figure 19, and the corresponding findings are presented in Table 10 for comprehensive analysis.

Whether the system falls within the unstable range can be determined by observing the voltage values. In Figure 19, a fixed magnitude of the external force was maintained while adjusting the input frequency of the system. Consider the experimental measurement in Figure 19c as an example. Initially, the magnitude of the external force was set at 0.4, and the system frequency was adjusted to 16.2 Hz, corresponding to the first mode. Consequently, the system entered the unstable region, and the voltage was measured for 15 s in this state. Subsequently, the system frequency was adjusted to 13 Hz, placing the system within the stable region, and the voltage was measured again for 15 s. The results clearly illustrate that the voltage of the system experiences a significant drop in the stable region, confirming the theoretical prediction shown in Figure 19b. This correlation between system stability and voltage generation efficiency is evident from the experimental measurements. In the study by Mei et al. [27], a comparison of recent clamped–clamped energy harvesters’ key characteristics was conducted. Resonant frequencies range from 27 to 70 Hz, with voltage and power output varying from 0.028 V to 4.05 V and 0.08 μW~1.9 μW, respectively. Power densities span from 2.68 × 10^−3^ μW/mm^3^~3.73 × 10^−2^ μW/mm^3^. The present investigation achieved a maximum voltage and power output of 3.83 V and 0.222 mW with slapping force (as shown in Table 5), resulting in a power density of approximately 0.66 μW/mm^3^. Parametric excitation enabled diverse modal frequencies, effectively enhancing power generation across different ranges. Notably, this design offers a broader bandwidth advantage over traditional devices, as illustrated in Figure 19, thus showcasing the benefits of this study.

To address the concern regarding piezoelectric patch brittleness, several measures were taken to ensure its reliability and durability. Firstly, piezoelectric materials known for their mechanical robustness and flexibility were carefully selected, enhancing their capacity to withstand potential impacts and stress. In this experiment, PZT-grade PZT-5H, a widely recognized flexible piezoelectric material, was employed. The experiment was repeated five times, with each slap lasting five minutes. Importantly, even after a brief period, no decrease in voltage output was observed, highlighting the durability of PZT-5H within these specific conditions and its suitability for this application. Secondly, during the integration process, the PZT was meticulously affixed to ensure proper attachment to the elastic steel while minimizing stress concentrations. This research introduces the concept of slapping and includes experiments that validate its feasibility. For the selection of future PZT materials or the potential addition of protective devices to the PZT, these avenues offer the opportunity to enhance PZT’s real-time protection. These considerations provide valuable directions for subsequent research in this field.

Based on the findings presented in Table 10, it is evident that when the external force is fixed at 0.4 and the system frequency is set to 16.2 Hz for the first mode and 28.8 Hz for the second mode (resulting in the system being within the unstable region), the voltage generation efficiency is notably high, reaching an output of above 3 volts. Conversely, when the system frequency is set to 13 Hz, 19 Hz, 27.5 Hz, or 30 Hz (placing the system in the stable region), the voltage generation efficiency experiences a significant drop. The error between the theoretically calculated values and the experimentally measured values is within 10%, further confirming the accuracy of predicting the unstable range of parametric excitation. This result once again validates the correctness of the theoretical predictions with respect to the unstable range of parameter excitation. The results of this study underscore the novelty of the present approach. By comparing the voltage generation benefits of the first and second modes, light is shed on the un-slapped potential of the latter in vibration energy harvesting systems. Moreover, the incorporation of the baffle introduces an unprecedented boost in voltage generation, clearly demonstrating the innovative edge of this proposed methodology. These findings reinforce the originality of this research and its potential to revolutionize energy harvesting efficiency. This study additionally investigates the frequency response of parametric excitation, extending the resonant frequency range around the linear natural frequency (Figure 19). This expansion contributes to a broader usable bandwidth compared to conventional designs.

## 7. Conclusions

This study delved into the analysis of parametric excitation in a nonlinear elastic beam with fixed–fixed (roller) boundary conditions. The equation of motion was derived using Newton’s Second Law, Euler’s angle transformation, and the Taylor series, within the framework of nonlinear beam theory. Employing the method of multiple scales (MOMS), the phenomenon of parametric excitation was investigated. Verification of the presence of unstable regions was achieved through the generation of fixed-point plots, time–response plots, and phase plots. These plots affirm the accuracy of pinpointing unstable regions. Furthermore, this study integrated the piezoelectric equation with the nonlinear equation, enabling exploration of the nonlinear system’s unstable regions under a variety of frequencies and external forces. This exploration aimed to determine the maximum voltage efficiency achievable. In the final phase, a baffle was introduced into the system to impart a slapping force onto the Piezoelectric (PZT) element. Through this addition, we aimed to assess the potential enhancement of power conversion benefits. This comprehensive investigation yielded the following conclusions:

1. The system’s parametric excitation characteristics allow for vibration control by altering the frequency of the nonlinear elastic beam system or adjusting the magnitude of the external force. To analyze the maximum amplitude point of the system, fixed-point plots were employed, while time–response and phase plots were utilized to validate the accuracy of the results obtained.
2. By examining the stable and unstable regions in the parameter plane through diagrams, the system’s stability can be determined. When the system falls within the unstable region, a larger amplitude is exhibited, resulting in higher power generation efficiency. Consequently, the assessment of whether the system resides in the unstable region is accomplished by experimentally measuring the voltage magnitude, as well.
3. To achieve the optimal electric power output from the system, the internal resistance value was calculated to be 66 K ohms. Both theory and experiments showed that better power generation benefits were yielded by the second mode compared to the first mode. This was attributed to the higher impacting frequency of the second mode, resulting in increased electric power generation efficiency compared to when using the first mode. While the theoretical voltage was higher than the experimental voltage, the discrepancy remained within an acceptable range of 10%. Furthermore, the addition of a baffle (slapping force) further enhanced the voltage generation benefits, leading to even higher power conversion efficiency.
4. The conventional vibration energy harvesting system utilized the vibration deformation of an elastic beam to generate electricity. In this research, a slapping force was introduced to enhance the voltage generation efficiency. The voltage generation output was evident, showcasing significant potential in the future industrial market for wireless sensors or microelectro-mechanical system structures.

This study presents a novel perspective on vibration energy harvesting, focusing on precise positioning and enhancement techniques. By highlighting the advantages of the second mode, revealing the benefits of the added baffle, and expanding the bandwidth of the resonant vibration region, this work contributes substantially to current knowledge in this area. The unique insights and inventive methodologies showcased here hold promising potential for advancing energy generation systems in various sectors.

## Figures and Tables

**Table 1:** Dimensionless root mean square value with no additional slapping force.

	PZT at Max. Amp.	PZT at Root.
first mode	0.4248	0.4075
second mode	0.4778	0.4129

**Table 2:** RMS value of dimensionless voltage with additional slapping force.

	Max Amp.	1/2 Max Amp.
first mode	0.4988	0.4211
second mode	0.5002	0.4857

**Table 3:** Voltages and powers for different load resistances.

		KΩ				
	Open Circuit	36	46	56	66	76
Case 1 (V)	3.622	1.228	1.4983	1.629	1.827	1.90
Case 2 (V)	3.628	1.2236	1.459	1.6668	1.857	2.028
Average (V)	3.625	1.2258	1.4787	1.6479	1.842	2.0225
Power (mW)	—	0.0417	0.0475	0.0485	0.0514	0.0508

**Table 4:** Experimental voltage and power output without slapping force.

	First Mode		Second Mode	
	Max Amp.	Root	Max Amp.	Root
Voltage (V)	1.9096	1.8296	2.7845	2.6407
Power (mW)	0.057	0.052	0.117	0.106

**Table 5:** Experimental voltage output with the slapping force.

	First Mode		Second Mode	
	Max Amp.	1/2 Max Amp.	Max Amp.	1/2 Max Amp.
Voltage (V)	2.7412	2.4984	3.8263	3.5596
Power (mW)	0.114	0.095	0.222	0.192

**Table 6:** Comparison of dimensioned theoretical and experimental voltage values without slapping force.

	First Mode		Second Mode	
	Max Amp.	Root	Max Amp.	Root
Theo. (V)	2.0235	1.9247	3.0403	2.8710
Exp. (V)	1.9096	1.8296	2.7845	2.6407
Error (%)	5.63	4.92	8.41	8.02

**Table 7:** Comparison of dimensioned theoretical and experimental voltage values with slapping force.

	First Mode		Second Mode	
	Max Amp.	1/2 Max Amp.	Max Amp.	1/2 Max Amp.
Theo. (V)	2.8703	2.6777	4.0654	3.8689
Exp. (V)	2.7412	2.4984	3.8263	3.5596
Error (%)	4.71	7.18	6.25	8.68

**Table 8:** Comparison of experimental voltage output of the system with/without slapping force.

	Max Amp.	
	First Mode	Second Mode
No slap (V)	1.9096	2.7845
Slap (V)	2.7412	3.8263
Increase (%)	43.55	37.41

**Table 9:** Comparison of theoretical and experimental root mean square values of the unstable region of the system under different external forces.

Input FrEq.	13.8 Hz		17.8 Hz		28 Hz		29.5 Hz	
Ext. Force	0.4	0.3	0.4	0.3	0.4	0.3	0.4	0.3
Theo. (V)	1.5006	1.2491	2.5302	1.9616	3.3838	0.2072	3.5125	0.1375
Exp. (V)	1.4454	1.1806	2.3971	1.8999	3.1409	0.1901	3.2235	0.1242
Error (%)	3.68	5.49	5.26	3.15	7.18	8.25	8.23	9.72

**Table 10:** Comparison of theoretical and experimental root mean square values of the unstable region of the system at different frequencies.

	External Force ε=0.4							
Input FrEq.	16.2	13	16.2	19	28.8	27.5	28.8	30
Theo. (V)	1.6967	0.8997	1.6967	0.7504	3.2076	2.3210	3.2076	1.3783
Exp. (V)	1.6039	0.8587	1.5549	0.7066	3.0251	2.1551	3.0664	1.2554
Error (%)	5.47	4.56	8.35	5.84	5.69	7.15	4.40	8.92

## Data Availability

Not available.
